# Effect of drug metabolism in the treatment of SARS-CoV-2 from an entirely computational perspective

**DOI:** 10.1038/s41598-021-99451-1

**Published:** 2021-10-07

**Authors:** João Paulo Almirão de Jesus, Letícia Cristina Assis, Alexandre Alves de Castro, Elaine Fontes Ferreira da Cunha, Eugenie Nepovimova, Kamil Kuca, Teodorico de Castro Ramalho, Felipe de Almeida La Porta

**Affiliations:** 1 Laboratory of Nanotechnology and Computational Chemistry, Federal Technological University of Paraná, Avenida dos Pioneiros 3131, Londrina, Paraná CEP 86036-370 Brazil; 2 Department of Chemistry, Federal University of Lavras, Lavras, Minas Gerais CEP 37200-000 Brazil; 3 Department of Chemistry, Faculty of Science, University of Hradec Kralove, Rokitanskeho 62, 500 03 Hradec Králové, Czech Republic

**Keywords:** Viral infection, Computational chemistry

## Abstract

Understanding the effects of metabolism on the rational design of novel and more effective drugs is still a considerable challenge. To the best of our knowledge, there are no entirely computational strategies that make it possible to predict these effects. From this perspective, the development of such methodologies could contribute to significantly reduce the side effects of medicines, leading to the emergence of more effective and safer drugs. Thereby, in this study, our strategy is based on simulating the electron ionization mass spectrometry (EI-MS) fragmentation of the drug molecules and combined with molecular docking and ADMET models in two different situations. In the first model, the drug is docked without considering the possible metabolic effects. In the second model, each of the intermediates from the EI-MS results is docked, and metabolism occurs before the drug accesses the biological target. As a proof of concept, in this work, we investigate the main antiviral drugs used in clinical research to treat COVID-19. As a result, our strategy made it possible to assess the biological activity and toxicity of all potential by-products. We believed that our findings provide new chemical insights that can benefit the rational development of novel drugs in the future.

## Introduction

Since the start of the last year, the novel coronavirus SARS-CoV-2, which is responsible for the disease designated like Coronavirus Disease 2019 (abbreviated as, COVID-19), has led to the death of over 2.7 million peoples^1–12^. Moreover, the pandemic significantly impacted regular operations and economies of several countries, ultimately affecting millions of people both directly and indirectly. To combat the disease, the scientific community is developing vaccines and medicines; however, to date, there is no proven effective treatment for COVID-19^1–9^. Considering the increasing global caseload, there is substantial pressure to discover and develop new antiviral drugs and vaccines to treat COVID-19.

Recent studies suggest that the structure of the SARS-CoV-2 virus is suitable for hosting interactions between its active sites and other molecules. The current focus of new drug treatments is to target the essential areas of the virus, including the Spike (S) protein, 3C-like protease (3CLpro), papain-like protease (PLpro), RNA-dependent RNA polymerase (RdRp), and also serine protease TMPRSS2^2–7^. Molecules that show favourable interactions with the active sites of these proteins may inhibit their enzymatic activities and hinder the essential mechanism of viral pathogenicity. Possible candidates for the treatment of COVID-19 include the antiviral drugs Favipiravir, Galidesivir, Nitazoxanide, Remdesivir, Ribavirin, Chloroquine, and Hydroxychloroquine due to their ability for the enzymatic inhibition of SARS-CoV-2^7–13^.

In addition to the effectiveness of a particular drug in the treatment of a disease, drug design must also consider xenobiotics, or the mechanism via which the by-products of a medicine interact with and exit the human body^14–17^. As is well known, the general mechanism of xenobiotics involves three main phases: Phase I, drug activation through oxidation, reduction, and hydrolysis; Phase II, drug inactivation by means of conjugation with proteins to yield water-soluble metabolites that can be eliminated from the body; and Phase III, biotransformation by enzymes before the waste leaves the body^14–17^. However, predicting the details of this effect remains a challenge.

The present work proposes a theoretical methodology to predict the potential by-products of the abovementioned drugs used to treat COVID-19. This novel method simulates the electron ionization mass spectrometry (EI-MS) fragmentation of the drug molecules used to treat COVID-19 and evaluates the structures of the intermediates to explain the possible xenobiotic metabolism for each species. These results revealed two different molecular docking models to inhibit the main protease (M^pro^) and RdRp of the SARS-CoV-2 virus. In the first model, the drug is docked without considering the possible metabolic effects. In the second model, each of the intermediates from the EI-MS results is docked, and metabolism occurs before the drug accesses the biological target. To the best of our knowledge, this is the first study to present an entirely theoretical approach for modelling drug metabolism in the treatment of COVID-19. Therefore, we present a novel methodology that may contribute to the design of new drugs.

## Computational models and methods

All structures were fully optimized and confirmed as a minimum of potential energy by means of the Density Functional Theory (DFT) method at the B3LYP level with the 6–31 + G(d,p) basis set. These optimized structures were then used in all subsequent calculations, including energy calculations were carried out through Time-Dependent DFT (TD-DFT). All of DFT and TDDFT calculations were performed on Gaussian 09 package^18^. Then, the trajectories of fragmentation and computed EI-MS spectrum for the main antiviral drugs used in COVID-19 treatment were predicted by the QCEIMS program^19,20^, and these results were visualized in Grace software (https://plasma-gate.weizmann.ac.il/Grace/). For these simulations, two semiempirical methods (GFN1-xTB and GFN2-xTB) were initially considered for the Chloroquine and Hydroxychloroquine drugs and compared with experimental data^20^. In both cases, the GFN2-xTB method better reproduces experimental data, as shown in Fig. S1 of supplementary material, being therefore chosen for the calculations of all compounds investigated in this study. In all cases, the total simulation time was 5 ps, initial temperature of the vaporized substract of 500 K, and impact excess energy of 0.6 eV atom^−1^^19,20^. From the computed EI-MS spectrum were identified the intermediaries' structures and plotted in the Avogadro code^21^. All 2D structures were draw in the MarvinSketch 20.10 software (https://chemaxon.com/products/marvin).

**Figure 1:**
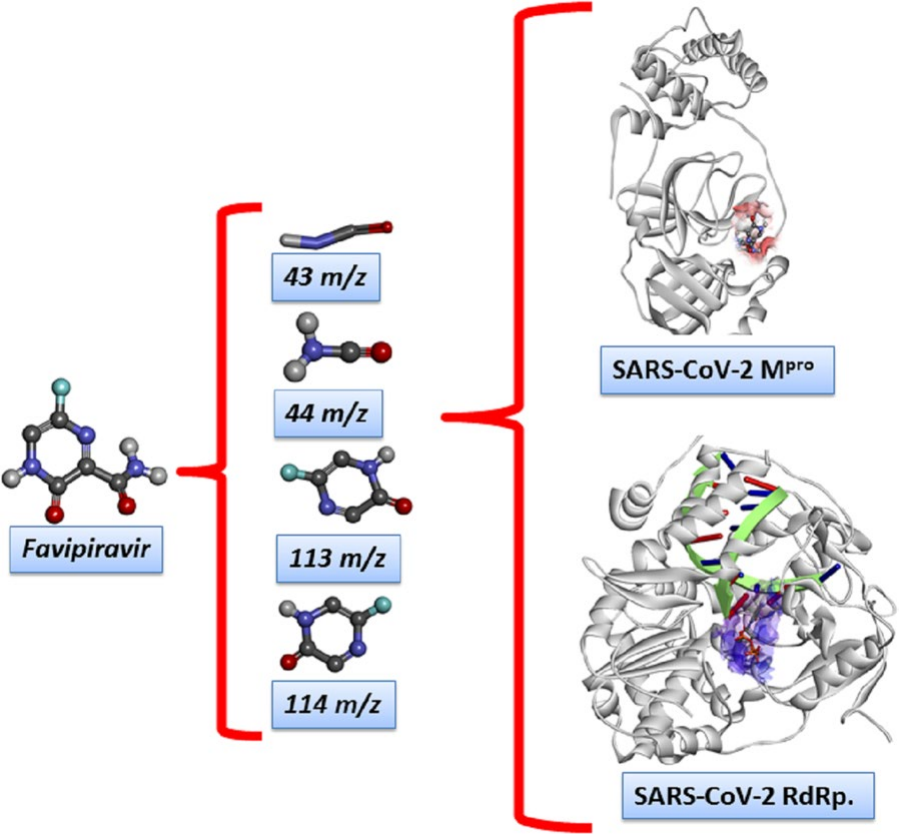
Schematic representation of computational strategy proposed in this study to the investigation of the metabolism effect in drug design. The strategy used to build these models have based on three different situations: (**a**) no previous metabolism and (**b**) prior effect of metabolism to the inhibitory process. Image generated in the Discovery Studio Software 4.5: https://discover.3ds.com/discovery-studio-visualizer-download.

Three different molecular docking models were prepared in this study as strategy to investigate the possible metabolic effects, as shown in Fig. 1, and all these calculations were performed with the tool AutoDock Vina (version 1.1.2)^22^, as implemented in the MolAr (Molecular Architecture) software^23^. The crystallographic M^pro^ and RdRp structures used in this simulations were prepared according to our previous studies^12,13,24^. Based on this strategy, we also investigate the absorption, distribution, mechanism, excretion, and toxicity (ADMET) properties for antiviral target drugs and their by-products metabolic. Thereby, the acute toxicity and other relevant pharmacokinetics parameters can easily be obtained from a rat model-based admetSAR predictor (http://biosig.unimelb.edu.au/pkcsm/prediction). This ADMET procedure has been used previously in similar systems with large success^12,13,25^.

In a further theoretical insight, the key docking complexes of the M^pro^ enzyme were evaluated by molecular dynamics (MD) simulation (at 300 K and at 1 bar) using the GROMOS54A7 all-atom force field^26^ and performed using GROMACS 5.1 software^26,27^. First, the M^pro^ complex has inserted into a 12 Å water (periodic boundary) box with the SPC solvation model that incorporates sodium and chlorine ions for a complete charges neutralization and electrostatic interactions were treated using the Particle Mesh Ewald method. About 5000 cycles were used for minimized the complexes and then were submitted to a total simulation time of 10 ns. MD trajectories were analyzed in the Visual Molecular Dynamic (VMD, version 1.9.3) program^28^.

## Results and discussions

In this work, we proposed a novel strategy to predict the metabolism of the possible by-products of the drugs cited in the introduction session.

As seen in the Fig. S5a, the EI-MS diagram from the GFN2-xTB method, in principle, presented most of the intermediaries of the Chloroquine molecule, in agreement with the experimental data profile (NIST MS 42361), with slight deviation of intensity. The most intense signal 86 m/z and its respective intermediary are also identified in the theoretical plot and the resultant trajectory described. As for the Hydroxychloroquine spectra in Fig. S5b, the most intense peaks from the experimental data (NIST MS 246973) are not identified in the theoretical data, still, some of the signals are found with distinct intensity from the experimental spectra. In order to evaluate the results between the theoretical methods, Fig. S5c shows the EI-MS spectra of the Chloroquine obtained using the GFN1-xTB method in comparison with the same experimental profile. In this manner, the spectra from the GFN1-xTB did not match the intermediaries mass/charge rate and intensity as well as its analog method, reveling that the GFN2-xTB is the best option for this calculation. Transitioning to the Hydroxychloroquine in Fig. S5d, the spectra acquired from the GFN1-xTB approach did not show significant improvements over the GFN2-xTB, and the most intense signals of the NIST profile are not identified as well. In general, an increasing on the molecular dynamics parameters could lead to a better prediction of the EI-MS spectra and its intermediaries in exchange of meaningful computational cost, however, as the current methodology with GFN2-xTB provided satisfactory results for the Chloroquine drug, it has been chosen as the default semiempirical method to the study of the other drugs.

Henceforth, the discussion of the EI-MS spectra and trajectories will be done in the context of xenobiotics metabolism, evaluating the obtained intermediaries as drug by-products, their metabolism and toxicity when possible. Thus, returning to the Chloroquine drug, the spectra and trajectories are shown in Fig. S5a. The Chloroquine molecule contains polar amine and chloride groups in its structure, showing an aromatic region with more polar character than the other extremity. The first trajectory showed the fragmentation of Chloroquine around the amine that bond the aromatic and the alkane regions, leading to the following intermediaries: the I-177 m/z 7-chloro-4-aminoquinoline, containg the aromatic region, deprotonated amine and chloride polar groups, is a toxic and major metabolite from the oxidation of Chloroquine by the cytochrome P-450 enzyme^29,30^; the deprotonated I-57 m/z butane and I-29 m/z ethane, both nonpolar hydrocarbons which can be oxidized into polar species in Phase I of metabolism; and the I-56 m/z deprotonated amine, a polar and likely water-soluble molecule that may metabolize directly in Phase II. The trajectory II leads to the high molecular mass fragment II-233 m/z similar to the I-177 m/z, with an alkane extremity that may be target of oxidation in Phase I; and the II-86 m/z, the specie also identified in the experimental spectra, show very low polar character and may be almost insoluble in water, possible target of oxidative reactions in Phase I metabolism before conjugation in Phase II. The last trajectory for Chloroquine gives the following intermediaries: the III-205 m/z, a specie like the I-177 m/z and II-233 m/z, with a shorter alkane segment which may be oxidized in Phase I, and share the behavior of its analog molecules; the deprotonated organic molecules III-29 m/z ethane and III-28 m/z ethene, both nonpolar and likely targets to oxidizing reactions in Phase I, leading to polar conjugates to metabolize in Phase II; and the protonated form of I-56 m/z.

Figure S5b shows the EI-MS spectra and unique trajectory of the Hydroxychloroquine drug. The molecular structure of this drug is a more polar analog of the Chloroquine due to the addition of a hydroxide group. The calculations for the Hydroxychloroquine resulted in a single trajectory: the I-142 m/z, a deprotonated aminoquinoline similar to the 7-chloro-4-aminoquinoline from the metabolization of Quinoline, which is a metabolite from the Hydroxychloroquine^31^; the I-35 m/z chloride ion; and the I-144 m/z, with polar amine and alcohol groups, and the nonpolar extremities likely submitted to oxidative reactions in Phase I that may lead to smaller and polar fragments. This last fragment is further cleaved into two more species: the I-1–31 m/z molecule, which is deprotonated into a highly water-soluble and toxic formaldehyde form, being rapidly metabolized into formate by the alcohol dehydrogenase enzyme^32,33^; and the I-1–113 m/z, an amine with pentane and ethane extremities, and a possible target for Phase I oxidative reactions that have as products smaller and polar molecules, further being transformed into metabolites in Phase II.

**Figure 2:**
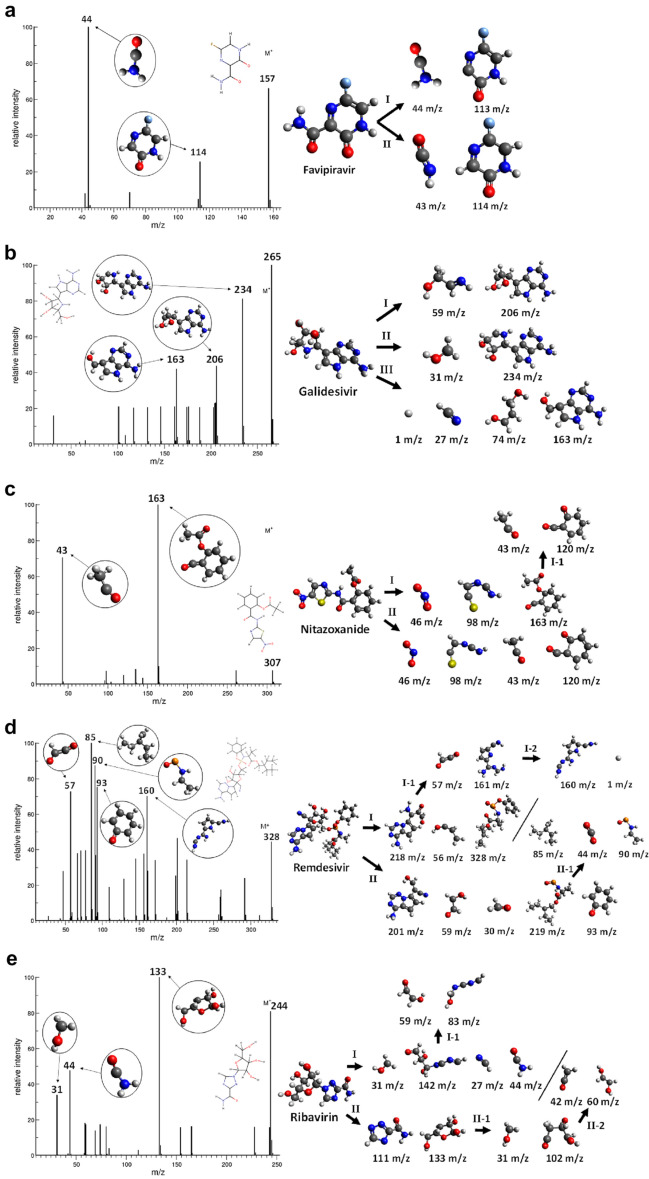
EI-MS fragmentation spectra and trajectories of (**a**) Favipiravir, (**b**) Galidesivir, (**c**) Nitazoxanide, (**d**) Remdesivir and (**e**) Ribavirin.

As shown for the Chloroquine and Hydroxychloroquine, the theoretical EI-MS calculations done with our methodology were able to predict the main metabolites of these drugs, the 7-chloro-4-aminoquinoline and aminoquinoline, respectively. In this framework, we suggest a possible metabolism pathway for the Favipiravir, Galidesivir, Nitazoxanide, Remdesivir, and Ribavirin drugs, as they are receiving substantial attention due to the SARS-CoV-2 outbreak. In our previous work, we estimated the EI-MS spectra and identified the possible trajectories for the Favipiravir molecule and its tautomers^12^. In the current work, we expanded our analysis to evaluate each intermediary species' biochemical activity. The computed EI-MS spectrum of Favipiravir has two main trajectories that yield similar species (Fig. 2a). The fragments shown in Trajectory I are the unstable analogues of Trajectory II; thus, the existence of the II-43 m/z and II-114 m/z species is the most probable mechanism. The fragments in Trajectory I are easily transformed into Trajectory II via the exchange of protons between the molecules due to the difference in charge. Both fragments obtained in Trajectory II are polar and water-soluble due to hydrogen bonds in the C=O, N–H, and C=N groups, respectively^34,35^. Therefore, the Favipiravir waste molecules can be directly transformed into metabolites through conjugation with proteins in Phase II of the metabolism.

Figure 2b shows three trajectories for Galidesivir. The structure of Galidesivir includes several polar groups showing two extremities with alcohol and amine groups. Consequently, the molecular structure is fragmented around the alcohol extremity, resulting in a main cyclic molecule, including the amine extremity and other smaller linear by-products with hydroxide groups. The heavier fragments I-206 m/z, II-234 m/z, and III-163 m/z consist of several polar groups, including O–H, N–H, N–H_2_, and C=N. These are soluble in water due to hydrogen bonds and are efficiently metabolized in Phase II. It is possible that Phase I hydrolysation can also occur in the O–H and N–H groups, leading to smaller polar fragments. Regarding the smaller by-products of Galidesivir, the I-59 m/z fragment is easily transformed into a metabolite due to its polar O–H and N–H groups. The II-31 m/z fragment is unstable in its present form, and it transfers a proton to its main molecule to adopt a formaldehyde form. The III-74 m/z fragment is stable and water-soluble due to the diol group in its extremity. However, the III-27 m/z fragment is hydrogen cyanide, a water-soluble and extremely poisonous molecule. It is well known for its inhibition of oxidative phosphorylation through binding with the cytochrome enzyme, halting the aerobic metabolism^36^. The III-1 m/z fragment is a proton, which is unlikely to exist in this form and may bond to its respective III-163 m/z molecule. The results show that all the waste molecules from the alcohol extremity of Galidesivir are directly metabolised in Phase II, without the need for Phase I transformations. However, due to the possible toxic by-products, we encourage further studies of this molecule from an experimental perspective.

The computed EI-MS spectra and trajectories for Nitazoxanide are shown in Fig. 2c. The molecular structure of this drug includes multiple polar functional groups, such as nitro, sulphide, amine, amide, and ester. Nitazoxanide fragmentation predominantly occurs at the amide carbonyl, pentacyclic carbon, and nitro groups, resulting in three main species. The I-163 m/z fragment is polar (due to the ester and carbonyl groups) and highly nucleophilic due to the carbonyl group, and it most probably exists in a protonated form, and hence expected to be oxidized in Phase I of metabolism. The I-98 m/z fragment is nucleophilic (due to two amines and one sulphide groups) and probably exists in an isomer form due to proton transfer among the N–H and the C–S extremity, both with significant polarity. The I-46 m/z fragment is a polar and highly reactive nitro radical that quickly transforms into other species. The I-163 m/z fragment may separate into two nucleophilic by-products, the I-1–120 m/z and I-1–43 m/z fragments, both of which are polar and easily protonated. In particular, the I-1–120 m/z fragment stabilization through proton acceptance, likely to be moderately water-soluble due to its polar carbonyl and ketone groups, and may suffer oxidative reactions in Phase I metabolism before its synthesis step. This results in alcohol in the case of the I-1–120 m/z fragment. While the I-1–43 m/z fragment, is protonated into the water-soluble and toxic acetaldehyde in an acid environment, further metabolized into acetic acid by the aldehyde dehydrogenases enzyme^37^. The by-products are the same for Trajectory II. All the waste products attributed to nitazoxanide have significant polarity. Therefore, they can easily transform into metabolites during the xenobiotic metabolism processes.

The computed EI-MS spectrum and trajectories for Remdesivir are illustrated in Fig. 2d. The molecular structure of this drug is large and contains amine, alcohol, ether, ester, and diazo groups. Hence, Remdesivir is cleaved around the sulfur centre, leading to two major molecules, the I-218 m/z fragment (similar to the II-201 m/z fragment), I-328 m/z fragment (related to the II-219 m/z fragment), and other smaller fragments. Trajectory I resulted in three molecules: an I-218 m/z fragment with most of the amine, carbonyl, aldehyde (previously alcohol), and diazo polar groups; an I-328 m/z fragment with a polar PNO_2_^-^ centre, and ester groups and nonpolar extremities; and an I-56 m/z alcohol fragment, being directly metabolized in Phase II by synthesis with glutathione enzyme^38^. All these species are deprotonated during fragmentation and may be re-protonated in an acidic environment. In Trajectory I, the I-218 m/z can be further separated into two other polar molecules: the I-1–57 m/z fragment, which can be stabilized into an ethylene dione by means of deprotonation; however, experimental and theoretical studies suggest that this molecule is extremely short-lived and quickly dissociated into two CO molecules^39^, and the I-1–161 m/z fragment, which contains only amine groups due to the cleavage of the diazo group. As such, the I-2–160 m/z fragment is unlikely to exist. Instead, the I-1–161 m/z can be stabilized through intramolecular proton transfer, showing the possibility of hydrolyzation through these sites or direct metabolization in Phase II. The intermediates in Trajectory II are structurally similar to those of Trajectory I, resulting in the following deprotonated fragments: the II-201 m/z fragment, an analogue to the I-218 m/z fragment with polar amine, diazo, and alcohol groups; II-59 m/z fragment, a polar deprotonated diol; the II-30 m/z fragment, a polar and toxic formaldehyde; the II-219 m/z fragment, an unstable molecule similar to the I-328 m/z fragment without the phenolate ring and containing PON^2−^ and ester groups at its polar extremities; and the II-93 m/z fragment, a phenolate that may exist in a barely water-soluble and toxic phenol form^40^. Then, the II-219 m/z intermediary is posteriorly cleaved into three other by-products: the nonpolar II-1–85 m/z alkene, the nonpolar II-1–44 m/z carbon dioxide, and the polar and unstable II-1–90 m/z fragment with the PON^2−^ group. From these results, we can infer that most of the waste by-products of Remdesivir are polar species and can be transformed into metabolites via conjugation with the metabolism proteins. The II-93 m/z and II-1–85 m/z fragments, which are nonpolar or barely polar, are targets of oxidative reactions in Phase I, leading to water-soluble polar products that can be further transformed into metabolites in Phase II.

The data obtained in the EI-MS calculations of Ribavirin are shown by the Fig. 2e. The molecular structure of this drug contains alcohol, ether, diazo, amine, and amide groups along the molecule. Trajectory I shows a more fragmented path resulted from the electron ionization process than II: a I-31 m/z specie, that similar to the case of Galidesivir, can be further deprotonated into polar and toxic formaldehyde; the I-142 m/z with polar aldehyde, alcohol, and amine groups, which is unstable and may accept two protons, a likely water-soluble specie that may directly synthesize metabolites in Phase II metabolism; the I-27 m/z extremely poison and polar hydrogen cyanide, as also shown for Galidesivir; and the I-44 m/z, which can be further protonated into water-soluble formamide, a very important specie with role in the synthesis of nucleic bases, phosphorylation of nucleosides and other essential biological mechanism^41–43^. The I-142 m/z can be further dissociated into two more fragments: the I-1–59 m/z, accepting a proton and transforming into the same diol as shown for Remdesivir II-59 m/z; and the I-1–83 m/z, a deprotonated form with amine and alcohol groups and thus possibly water-soluble, likely metabolized in Phase II. Trajectory II leads to the II-111 m/z, a molecule with a polar nitrogenous ring and amide extremity, and the II-133 m/z, a polar oxygenated fragment with alcohol and ether groups, expecting appreciable water solubility and conjugation with proteins in Phase II of metabolism for both species. As such, the II-133 m/z fragment can be further cleaved, resulting in the II-1–31 m/z, lately being deprotonated into formaldehyde, and the II-1–102 m/z, a deprotonated polar molecule and possibly water-soluble due to its alcohol groups, also participating in Phase II. Additionally, the II-1–102 m/z fragment is after dissociated into II-2–42 m/z water-soluble and toxic ethenone^44^, and the II-2–60 m/z molecule, the same diol formed from Remdesivir II-59 m/z.

As such, the metabolism process for some relatively large molecules, yet unexplored by the literature, was predicted in this study and likely might be obtained by oxidation or hydrolysis reactions in Phase I or directly in Phase II. These results, however, showed various intermediary molecules with different toxicity levels. Hence, the metabolism study may give insight into the medication's possible counter-effects due to the metabolites' respective known reactions in the human organism.

### Docking results and ADMET

To investigate the interaction modes that our drug candidates performed with viral M^pro^ and RdRp of SARS-CoV-2 (PDB codes 5R82 and 3H5Y), respectively^45,46^. In order to assess the ability of the algorithm to predict likely ligand orientations, in particular, re-docking calculations were then performed in this study using the MolAr software^23^, with the implementation of the AutoDock Vina program^22^. As a result, it is important to note that the values extracted from RMSD (5R82 = 0.94 Ǻ/3H5Y = 1.55 Ǻ) showed that the program used in this study is adequate for predicting the conformation that the co-crystallized ligands adopted experimentally within the viral M^pro^ and RdRp of SARS-CoV-2^12,13,25^. The re-docking configurations were maintained to perform the docking calculations of the drugs investigated as well as their metabolism fragments. All computed interaction energy results are exhibited in Table 1.

**Table 1:** Interaction energy (in kcal mol^-1^) of drugs and their metabolism fragments computed through AutoDock Vina program.

Drugs	Metabolism fragments	Interaction energy (M^pro^/kcal mol^−1^)	Main interactions M^pro^	Interaction energy (RdRp/kcal mol^-1^)	Main interactions RdRp
Favipiravir		− 4.8	HIS164 ARG188	− 6.5	SER306 ASN309 ASP343 ARG182*
	44 m/z	− 1.9	HIS164	− 2.7	ASP343
	113 m/z	− 3.7	GLU166 ARG188*	− 4.8	G2 G8*
	43 m/z	− 2.0	–	− 2.8	–
	114 m/z	− 3.7	GLN189 HIS41* HIS164*	− 4.9	ASP247 G8
Galidesivir		− 3.9	HIS164	− 8.1	GLU168 G8
	59 m/z	− 2.4	MET165 GLN189	− 3.2	ASP343 ASP242 G8
	206 m/z	− 4.7	ARG188 GLN189	− 7.1	ARG182 G2
	31 m/z	− 1.5	HIS164	− 2.1	G8
	234 m/z	− 4.3	HIS164	− 7.7	GLU168 G8
	27 m/z	− 1.1	–	− 1.3	ASP343
	74 m/z	− 2.6	HIS164 MET165	− 3.5	ASP343
	163 m/z	− 4.6	MET165 ARG188	− 6.0	ASP343 ASN309
Nitazoxanide		− 5.6	GLN189 HIS164	− 7.9	ARG182 TRP246 ASP343 ASP242 ARG392
	43 m/z	− 1.8	HIS41	− 2.4	ASP343 G8
	120 m/z	− 4.0	GLU166 HIS41	− 5.0	ASP343 G8
	46 m/z	− 2.0	–	− 3.1	–
	98 m/z	− 2.8	ARG188	− 3.5	TRP246 ASN309 ARG182 ASP343
	163 m/z	− 4.3	GLU166	− 5.9	SER306 ASP343 ASN309 G8
Remdesivir		− 4.9	CYS145 MET49 GLU166	− 9.9	ASP343 G2
	57 m/z	− 2.1	HIS41	− 2.9	ASN309 ASP343 G8
	161 m/z	− 4.4	GLN189 HIS164	− 6.2	ASN309 ASP343 G8
	160 m/z	− 4.6	HIS164 ARG188	− 6.7	ASP343 G8
	218 m/z	− 4.1	CYS145 GLN189	− 7.4	ASN309 G8
	56 m/z	− 2.3	HIS164	− 3.2	ASP343
	328 m/z	− 3.4	HIS41 GLN189	− 7.6	TRP246 TYR243
	85 m/z	− 2.8	–	− 3.3	–
	44 m/z	− 1.7	HIS41	− 2.6	ASP343 G8
	90 m/z	− 2.5	HIS164 GLU166	− 3.4	G8
	201 m/z	− 5.0	ARG188	− 6.8	ASP247 ASP343 ARG182 G2
	59 m/z	− 2.3	–	− 3.0	G2
	30 m/z	− 1.3	HIS41	− 1.8	ASP343
	219 m/z	− 4.1	HIS164 GLU166	− 5.7	ASN309 TRP246 ARG245 ARG182
	93 m/z	− 3.5	–	− 4.5	ASP343 G8
Ribavirin		− 4.6	GLU166 HIS41	− 7.7	TRP246 TYR243 G2 G8
	59 m/z	− 2.3	GLN189	− 3.0	G8 G2
	83 m/z	− 2.8	HIS164 Glu166	− 3.9	ASP247 ASN309
	31 m/z	− 1.5	–	− 2.1	–
	142 m/z	− 4.0	GLN189 GLU166	− 5.3	ASP343 ASN309 G8 G2
	27 m/z	− 1.1	–	− 1.3	ASP343 G8
	44 m/z	− 2.0	HIS41 HIS164 MET165	− 2.7	–
	60 m/z	− 2.4	GLU166 GLN189 ARG188	− 3.1	ASP343 ASP247
	111 m/z	− 4.0	HIS164	− 5.2	G8 G2
	133 m/z	− 3.7	HIS164 GLU166	− 5.1	ASP343 TYR243 TRP246 G8
	102 m/z	− 3.2	GLN189 GLU166	− 4.3	ASP247 ASP343
Chloroquine		− 2.9	MET165* HIS41	− 6.9	G2
	29 m/z	− 1.3	–	− 1.6	–
	56 m/z	− 2.0	–	− 2.5	ASN309
	57 m/z	− 2.1	–	− 2.8	G2
	177 m/z	− 4.5	ARG188	− 6.3	–
	86 m/z	− 2.6	HIS41	− 3.1	–
	233 m/z	− 3.2	–	− 6.3	SER300
	28 m/z	− 1.3	–	− 1.6	–
	205 m/z	− 4.2	–	− 6.5	SER300
Hydroxychloroquine		− 3.2	–	− 7.3	ASP343
	142 m/z	− 4.6	GLU166	− 6.2	ASN309 G8
	144 m/z	–	–	–	–
	113 m/z	− 3.1	HIS164	− 3.9	–
	31 m/z	− 1.5	HIS164	− 2.1	ASP343

*Halogen bond-type interactions.

As shown in Table 1, all drugs investigated and their fragments stably interacted within the viral M^pro^ (with interaction energy values in the range of 0 to − 5.6 kcal mol^−1^) and RdRp sites (with interaction energy values in the range of 0 to − 9.9 kcal mol^−1^). In addition, the ADMET results are shown in Table 2.

**Table 2:** ADMET profile of diverse anti-viral drugs and their metabolism fragments.

Drugs	MS fragments	MW	logP	D.H/A.H	logS	Intestinal absortion (%)	CNS	Tox (LD_50_)
Favipiravir		157.104	− 0.992	2/3	− 2.103	81.635	− 3.111	1.929
	44 m/z	47.057	− 1.105	2/2	1.465	84.154	− 2.696	1.878
	113 m/z	114.079	− 0.091	1/2	− 0.781	94.787	− 2.932	2.131
	43 m/z	43.025	− 0.099	2/1	0.747	94.232	− 2.638	2.200
	114 m/z	116.095	− 0.517	1/2	− 0.001	100	− 2.996	2.208
Galidesivir		267.289	− 1.847	7/7	− 2.467	45.167	− 4.859	2.548
	59 m/z	59.068	− 0.372	2/2	0.967	85.397	− 2.910	1.996
	206 m/z	208.221	− 0.755	5/5	− 2.394	65.857	− 3.948	2.338
	31 m/z	32.042	− 0.392	1/1	1.075	98.165	− 2.566	2.029
	234 m/z	237.263	− 1.208	6/6	− 2.626	58.026	− 4.036	2.509
	27 m/z	27.026	0.140	1/0	0.162	100	− 2.375	2.351
	74 m/z	76.095	− 0.639	2/2	0.973	84.782	− 2.841	1.522
	163 m/z	170.216	− 2.148	4/5	− 1.142	65.04	− 3.959	2.081
Nitazoxanide		307.287	2.229	1/7	− 3.826	79.029	− 2.979	2.472
	43 m/z I	46.069	− 0.001	1/1	0.782	98.262	− 2.611	2.028
	120 m/z	122.123	0.663	0/2	− 0.501	100	− 2.681	1.862
	46 m/z	49.029	− 0.646	3/3	1.299	77.865	− 3.499	2.359
	98 m/z	106.194	− 0.578	3/3	0.821	81.893	− 2.996	2.244
	163 m/z	168.192	1.250	0/3	− 0.868	100	− 2.887	1.891
	43 m/z II	46.069	− 0.001	1/1	0.782	98.262	− 2.611	2.028
Remdesivir		602.585	2.312	13/13	− 3.56	43.813	− 5.006	2.213
	57 m/z	58.036	− 0.110	2/1	1.199	95.919	− 2.705	2.030
	161 m/z	166.208	− 2.321	8/4	− 1.112	56.409	− 3.974	1.992
	160 m/z	169.232	− 1.675	4/5	− 1.091	54.990	− 4.000	2.029
	218 m/z	223.236	− 2.129	3/7	− 1.957	63.682	− 3.675	2.226
	56 m/z	60.096	0.389	1/1	0.360	96.667	− 2.537	1.984
	328 m/z	329.333	3.123	2/4	− 3.279	89.124	− 2.817	2.511
	85 m/z	86.178	2.442	0/0	− 2.547	95.502	− 2.113	1.944
	44 m/z	47.057	− 1.105	2/2	1.465	84.154	− 2.696	1.878
	90 m/z	93.066	0.097	2/2	0.311	93.366	− 2.994	2.169
	201 m/z	203.205	− 0.089	2/6	− 2.983	76.079	− 3.111	2.222
	59 m/z	62.068	− 1.029	2/2	1.664	86.716	− 2.932	1.857
	30 m/z	30.026	− 0.185	1/0	0.722	100	− 2.393	2.040
	219 m/z	219.221	2.151	1/3	− 1.619	92.118	− 2.947	3.026
	93 m/z	98.145	1.520	1/0	− 0.963	97.244	− 2.739	2.040
Ribavirin		166.208	− 2.321	4/3	− 1.112	56.409	− 3.974	1.996
	59 m/z	60.052	− 0.882	2/1	1.156	95.474	− 2.743	1.846
	83 m/z	90.126	− 1.297	3/3	0.140	80.546	− 2.923	1.765
	31 m/z I	32.042	− 0.392	1/1	1.075	98.165	− 2.566	2.029
	142 m/z	144.130	− 1.406	2/4	0.04	77.520	− 3.167	1.914
	27 m/z	27.026	0.140	1/0	0.162	100	− 2.375	2.351
	44 m/z	47.057	− 1.105	2/2	1.465	84.154	− 2.696	1.878
	60 m/z	62.068	− 1.029	2/2	1.310	86.376	− 2.916	1.570
	111 m/z	112.092	− 1.096	2/3	− 1.065	73.849	− 3.903	1.900
	133 m/z	134.131	− 1.553	3/4	− 0.108	73.940	− 3.855	1.215
	31 m/z II	32.042	− 0.392	1/1	1.075	98.165	− 2.566	2.029
	102 m/z	104.105	− 1.071	2/3	1.030	81.777	− 3.420	1.378
Chloroquine		319.880	4.811	3/1	− 4.014	89.244	− 2.963	2.982
	29 m/z	30.070	1.026	0/0	− 0.623	100	− 2.344	2.182
	56 m/z	59.112	0.226	1/1	0.452	100	− 2.673	2.198
	57 m/z	57.096	0.707	1/0	− 0.156	100	− 2.505	2.277
	177 m/z	182.654	2.155	2/2	− 1.710	88.700	− 2.218	3.261
	86 m/z	87.166	0.958	1/0	− 0.303	100	− 2.807	2.173
	233 m/z	236.746	3.875	2/1	− 2.174	88.330	− 2.294	3.332
	28 m/z	30.070	1.026	0/0	− 0.623	100	− 2.344	2.182
	205 m/z	206.676	3.320	2/1	− 3.236	91.953	− 2.331	2.516
Hydroxychloroquine		321.852	3.740	4/2	− 3.347	89.139	− 2.194	2.770
	142 m/z	148.209	1.502	2/2	− 1.437	90.287	− 2.172	3.233
	144 m/z	145.246	1.488	2/1	− 0.765	92.423	− 2.900	2.123
	113 m/z	115.220	1.786	1/1	− 1.367	93.158	− 2.545	2.334
	31 m/z	32.042	− 0.391	1/1	1.075	98.165	− 2.566	2.029

ADMET parameters: *MW* molecular weight, *D.H* number of Hbonds donors, *A.H.* number of Hbonds acceptors, *logP* partition coefficient, *logS* predicted aqueous solubility (mol L^−1^), *CNS* predicted central nervous system, *Tox* Oral Rat Acute Toxicity (mol Kg^−1^).

Our ADMET analysis (Table 2) shows that the drugs Favipiravir and Chloroquine are more toxic than their main fragments. Remdesivir has a toxicity similar to its main fragments. While the Hydroxychloroquine, Galidesivir, Nitazoxanide and Ribavirin are less toxic than their fragments. Thus, this study points out the importance of verifying the effects of these pharmacophoric groups (fragments) for the contribution of developing new less toxic and more efficient drugs for the Covid-19 treatment.

### Favipiravir

**Figure 3:**
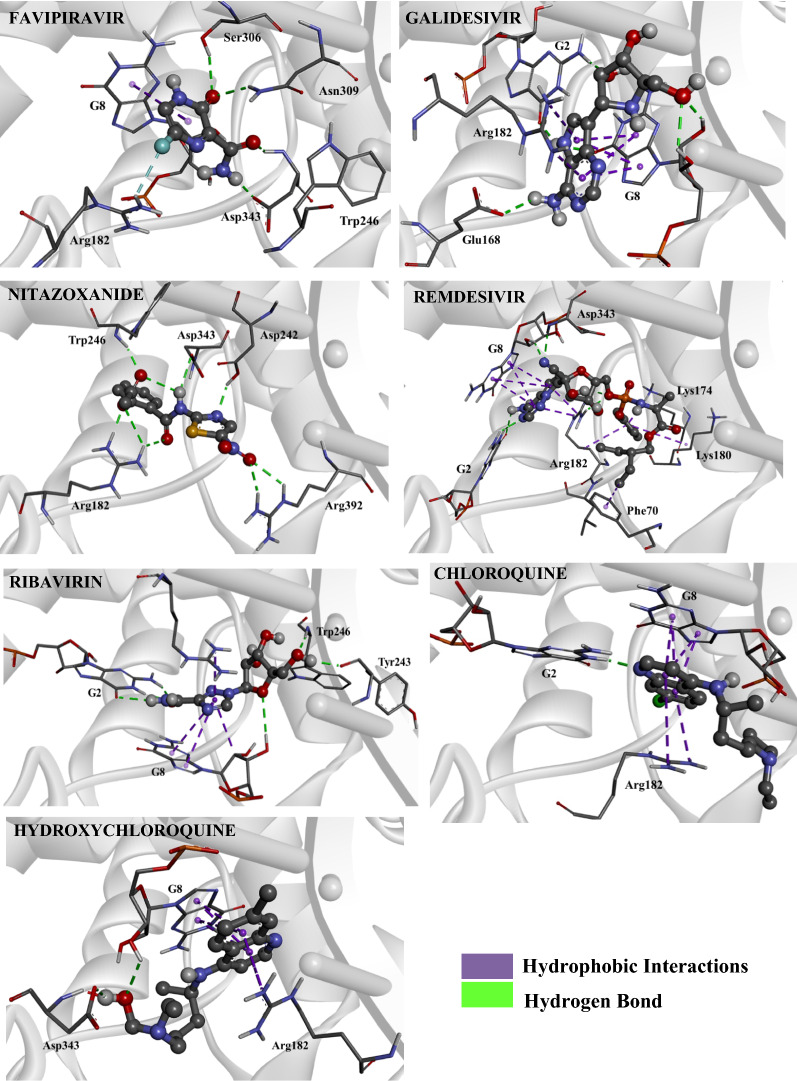
Intermolecular interactions of the drugs investigated in the RdRp binding site. Image generated in the Visual Molecular Dynamics 1.9.3 https://www.ks.uiuc.edu/Research/vmd/.

**Figure 4:**
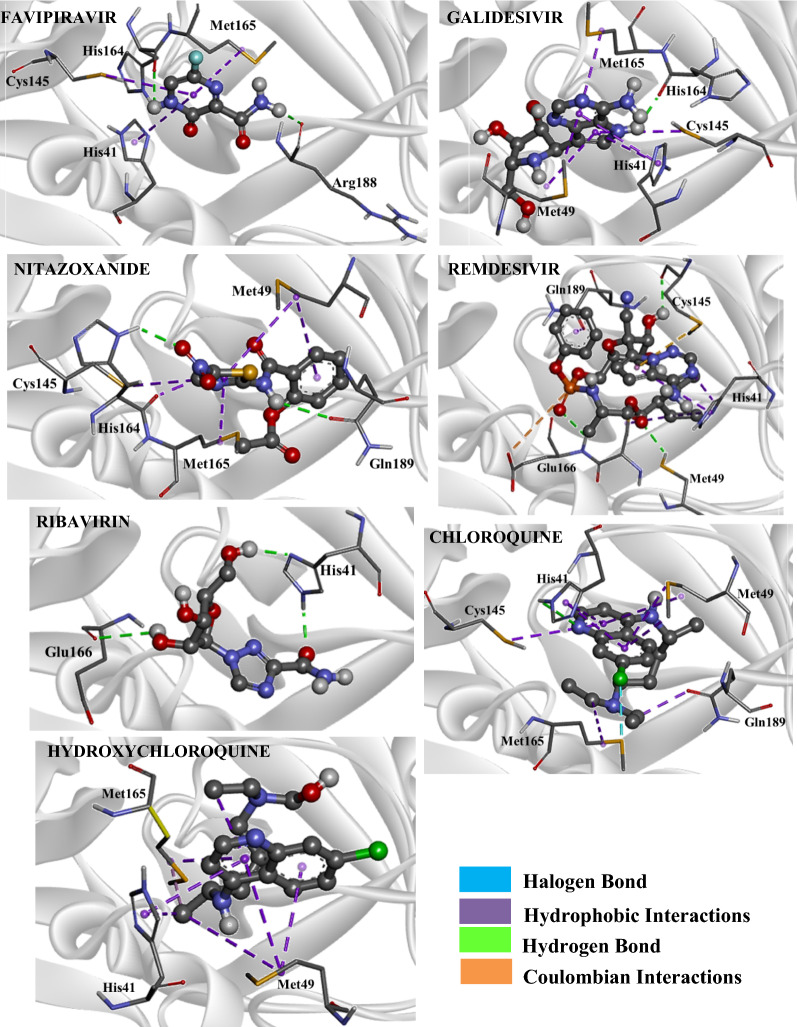
Intermolecular interactions of the drugs investigated in the M^pro^ binding site. Image generated in the Visual Molecular Dynamics 1.9.3 https://www.ks.uiuc.edu/Research/vmd/.

According to our results, Favipiravir showed a more stabilizing interaction energy (− 4.8 kcal mol^−1^) in both SARS-CoV-2 M^pro^ and RdRp binding sites, in comparison with its fragments. From our results, we can also notice that Favipiravir and its fragments had more stabilizing energies when docked in the RdRp binding site, in relation to our values found for the M^pro^ enzyme. This same trend can be observed for the other drugs investigated. Regarding the intermolecular interactions in the RdRp binding site, Favipiravir performed hydrogen bonds with the residues Asn309, Ser306, Asp343 and Arg182, and hydrophobic interaction with RNA (Fig. 3). In addition, in the M^pro^ binding site, there were interactions with the residues Met165, His164, Cys145, His41 and Arg188 (Fig. 4). In the RdRp binding site, as well as in the M^pro^ site, the fragments 113 m/z (M^pro^: − 3.7 kcal mol^−1^, RdRp: − 4.8 kcal mol^−1^) and 114 m/z (M^pro^: − 3.7 kcal mol^−1^, RdRp: − 4.9 kcal mol^−1^) showed the most stabilizing interaction energies in these target sites. These species also carried out the largest number of intermolecular interactions, like hydrophobic interactions and hydrogen/halogen bonds, as can be observed in Figs. S.6.1 and S.6.2 of supplementary material. According to the pharmacophoric graphs, the largest fragments of Favipiravir exhibited better interaction energies and a range of intermolecular interactions, probably due to the presence of more pharmacophoric groups in comparison with smaller fragments. In the RdRp site, it was observed different kinds of interaction with RNA from the fragments, such as hydrophobic interactions (fragments 43 m/z and 44 m/z), halogen bond (fragment 113 m/z) and hydrogen bond (fragment 114 m/z). From these results, we can observe that the bulkier fragments of Favipiravir more strongly interacted in the RdRp binding site. On the other hand, in the M^pro^ site, only the fragment 44 m/z interacted with both residues of the catalytic dyad (Cys145 and His41).

### Galidesivir

Regarding Galidesivir (M^pro^: − 3.9 kcal mol^−1^), the docking of its fragments exhibited three species with more stabilizing interaction energies in the M^pro^ binding site, in comparison with the non-metabolized drug. These species were the fragments 206 m/z (M^pro^: − 4.7 kcal mol^−1^, RdRp: − 7.1 kcal mol^−1^), 234 m/z (M^pro^: − 4.3 kcal mol^−1^, RdRp: − 7.7 kcal mol^−1^) and 163 m/z (M^pro^: − 4.6 kcal mol^−1^, RdRp: − 6.0 kcal mol^−1^) (Table 1). Our findings, however, indicate that these fragments have a better affinity in the binding site of M^pro^ than Galidesivir. On the other hand, in the RdRp, the non-metabolized drug showed a more stabilizing interaction energy than its fragments. In general, Galidesivir and its fragments showed a more stabilizing interaction in the RdRp binding site. By analysing the intermolecular interactions of Galidesivir in the RdRp site, this drug performed hydrogen bonds with Glu168 and RNA, and hydrophobic interactions with Arg182 and RNA (Fig. 3). In the M^pro^, Galidesivir carried out a hydrogen bond with His164, in addition to hydrophobic interactions with Met49, Met165, His41 as well as His164 (Fig. 4). The intermolecular interactions of its fragments can be observed in Figs. S.6.3 and S.6.4. In the RdRp binding site, it is interesting to notice that most fragments presented hydrophobic interactions with RNA. In addition, several fragments showed hydrophobic interactions with the catalytic dyad in the M^pro^ enzyme. These interactions are important for therapeutic activity^24^.

### Nitazoxanide

In this work, the interaction modes of a range of fragments of several drugs were analyzed toward the viral M^pro^ enzyme and RdRp, and in the case of Nitazoxanide, differently from Galidesivir, the non-metabolized drug showed more stabilizing interactions than all fragments obtained through QCEIMS, being these values − 5.6 kcal mol^−1^ for M^pro^ and − 7.9 kcal mol^−1^ for RdRp. The species with lower energies, that is, with more stabilizing energies, are the fragments 120 m/z (M^pro^: − 4.0 kcal mol^−1^, RdRp: − 5.0 kcal mol^−1^) and 163 m/z (M^pro^: − 4.3 kcal mol^−1^, RdRp: − 5.9 kcal mol^−1^). In general, when the non-metabolized drug is fragmented into larger fragments, these bulkier fragments often interact better in the binding site. Probably, this trend comes from the fact of these fragments present more pharmacophoric groups capable of performing intermolecular interactions in the target site. Note that this trend does not apply to all situations. For instance, another interesting fact is the possibility of some smaller fragments interact in a more stabilizing form due to the formation of charged atoms, favoring a specific kind of intermolecular interaction, that is, the well-known electrostatic interactions. This kind of interaction contributes to the total interaction energy of the ligand, in addition to the formation of stabilizing hydrogen bonds. Nitazoxanide performed hydrogen bonds with Arg182, Trp246, Asp343, Asp242 and Arg392 in the RdRp site, with no interactions with RNA (Fig. 3). In the M^pro^ active site, it was observed hydrogen bond interactions with Gln189 and His164, and hydrophobic interactions with Cys145, Met49 and Met165 (Fig. 4). According to the pharmacophoric graphs, the fragments carried out diverse intermolecular interactions. The fragments 43 m/z and 120 m/z interacted with RNA through hydrogen bonds, while the fragments 98 m/z and 163 m/z interacted through hydrophobic interactions (Fig. S.6.6). In the viral M^pro^ site, e.g. only the fragment 163 m/z interacted with both residues of the catalytic dyad (Fig. S.6.5). MD was carried for this ligand, within the viral M^pro^ enzyme, due to its most stabilizing interaction energy in the active cavity.

**Figure 5:**
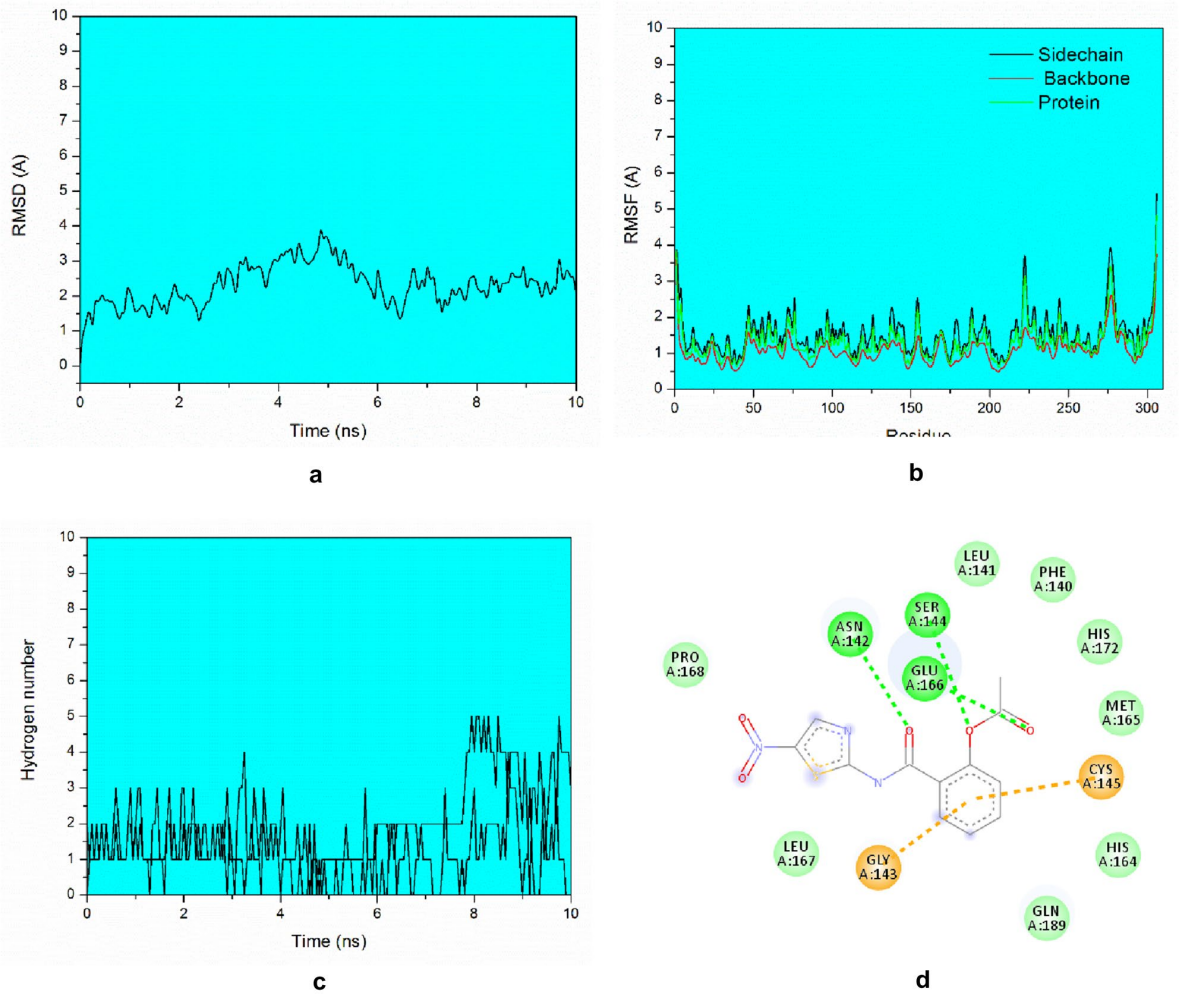
(**a**) 3 RMSD values of Nitazoxanide in the viral M^pro^ site monitored along 10 ns production phase in MD simulations. (**b**) RMSF values of Nitazoxanide. In the viral M^pro^ site obtained by average residual fluctuations over 10 ns MD simulation, analysis performed for the backbone, Protein and Sidechain. (**c**) Number of hydrogen bonds made by Nitazonamide at the Mpro site during molecular dynamics simulation. (**d**) Intermolecular interactions of the fragments of Nitazoxanide in the M^pro^ binding site. Green = hydrogen bond, Orange = hydrophobic interaction.

According to the RMSD values (Fig. 5a), it was observed that the drug Nitazoxanide oscillated a little within the active site (RMSD = 3Ǻ). This fact occurred because the structure of the ligand has several torsional angles, which led to an increase in the molecule's degree of freedom, thus taking a long time to reach equilibrium (8 ns) during the 10 ns of MD simulation. While the RMSF values (Fig. 5b) showed that the protein's main chain did not oscillate much, that is, the active site residues remained conserved inside the cavity. The biggest changes occur with residues Gln306, Gly275, Thr225 and Ser1 that are part of the side chain of the enzyme. Regarding interactions, the Hydrogen Bonding number (Fig. 5c) showed that the drug can perform up to three hydrogen bonds during the entire trajectory of MD. Analyzing in detail the interaction diagram (Fig. 5d), it was observed that the results corroborated the docking calculations, performing interactions with the residues His164, Gln189, Cys145 and Met 165, showing that the docking results were efficient in elucidating the interaction mode of this compound at the target site. Also, Nitazoxanide performed hydrogen bond interactions with key residues from the active site, remaining more reactive than the other compounds studied from the M^pro^ site.

### Remdesivir

For Remdesivir (M^pro^: − 4.9 kcal mol^−1^, RdRp: − 9.9 kcal mol^−1^), this antiviral resulted in a big amount of fragments from the study of metabolism. Note that some of its fragments showed interaction energies very close to that of the non-metabolized drug. These species were the fragments 161 m/z (M^pro^: − 4.4 kcal mol^−1^), 160 m/z (M^pro^: − 4.6 kcal mol^−1^) and 201 m/z (M^pro^: − 5.0 kcal mol^−1^), whose values highlighted here refer to the docking within the M^pro^ enzyme. In turn, Remdesivir was significantly more stabilized in the binding site of RdRp than its fragments. Remdesivir and its fragments showed more stabilizing interaction energies in the RdRp binding site, as can be observed in Table 1. Regarding the intermolecular interactions, Remdesivir performed hydrogen bond interactions with the residues Asp343 and RNA, and hydrophobic interactions with Lys174, Lys180, Phe70, Arg182 and RNA in the RdRp site (Fig. 3). As well, this drug interacted with Cys145, Met49 and Glu166 through hydrogen bonds in the M^pro^ site, along with Coulombian interactions with Cys145 and Glu166, and hydrophobic interaction with His41 (Fig. 4). Most fragments of Remdesivir interacted with RNA through hydrogen bonds and with some amino acid residues through hydrophobic interactions (Fig. S.6.7a and b). These fragments also showed a range of intermolecular interactions with diverse amino acid residues in the binding site. In the M^pro^ site, a lot of fragments interacted with the catalytic dyad through hydrophobic interactions and hydrogen bonds, they are the fragments 161 m/z, 160 m/z, 218 m/z, 56 m/z, 328 m/z, 201 m/z and 219 m/z (Fig. S.6.8a and b).

### Ribavirin

Another important antiviral investigated was Ribavirin. For this compound, the non-metabolized drug presented a more stabilizing interaction energy (M^pro^: − 4.6 kcal mol^−1^, RdRp: − 7.7 kcal mol^−1^) than its fragments. The species that best interacted with the biomacromolecules were the fragments 142 m/z (M^pro^: − 4.0 kcal mol^−1^, RdRp: − 5.3 kcal mol^−1^), 111 m/z (M^pro^: − 4.0 kcal mol^−1^, RdRp: − 5.2 kcal mol^−1^) and 133 m/z (M^pro^: − 3.7 kcal mol^−1^, RdRp: − 5.1 kcal mol^−1^). In the RdRp site, Ribavirin carried out hydrogen bonds with RNA and the residues Trp246 and Tyr243, in addition to hydrophobic interactions with RNA (Fig. 3). On the other hand, this drug interacted with only two residues in the M^pro^ binding site, being hydrogen bonds with Glu166, and one residue of the catalytic dyad, the residue His41 (Fig. 3). By analysing the pharmacophoric maps of the fragments (Figs. S.6.9 and S.6.10), most fragments stably interacted with RNA through hydrogen bonds. Furthermore, most fragments interacted with the residues of the catalytic dyad. This is an important finding, keeping in mind that the non-metabolized drug did not show interactions with the catalytic residue Cys145.

### Chloroquine

Going deeper into this investigation, and based on the results computed for Chloroquine with the viral M^pro^ enzyme, the fragments 177 m/z (M^pro^: − 4.5 kcal mol^−1^), 233 m/z (M^pro^: − 3.2 kcal mol^−1^) and 205 m/z (M^pro^: − 4.2 kcal mol^−1^) showed interaction energies more stable than that of the non-metabolized drug (M^pro^: − 2.9 kcal mol^−1^). Now taking into account the interaction modes obtained for the docking in the RdRp binding site, Chloroquine (RdRp: − 6.9 kcal mol^−1^) showed more stabilizing interaction energy than its fragments. The fragments 177 m/z, 233 m/z and 205 m/z exhibited interaction energy values close to that obtained for the non-metabolized drug. In the RdRp binding site, Chloroquine carried out hydrogen bond only with RNA, as well as hydrophobic interactions with RNA and the residue Arg182 (Fig. 3). In turn, in the M^pro^ binding site, Chloroquine interacted of different ways, such as through halogen bond with Met165, hydrogen bond with His41 and hydrophobic interactions with Cys145, Met165, Met49 and Gln189 (Fig. 4). Almost all fragments in the RdRp binding site interacted with RNA, except the fragments 28 m/z and 29 m/z (Fig. S.6.11). From these results, we can observe that the small fragments without hydrogen bond donor interacted less with DNA. In the M^pro^ binding site, most residues interacted with the residues of the catalytic dyad (Fig. S.6.12).

### Hydroxychloroquine

For Hydroxychloroquine, the fragment 142 m/z (M^pro^: − 4.6 kcal mol^−1^) was significantly more stable than the non-metabolized drug (M^pro^: − 3.2 kcal mol^−1^), based on the results acquired from the docking with the viral M^pro^. On the other hand, like Chloroquine, Hydroxychloroquine showed an interaction energy more stable in the RdRp binding site than those of its fragments. The fragment 144 m/z did not present any results. In the target site of RdRp, the fragments interacted with RNA through hydrophobic interactions, and only the fragment 142 m/z performed hydrogen bond with RNA (Fig. S.6.13). In the M^pro^ binding site, only the fragment 142 m/z interacted with both residues of the catalytic dyad, through hydrophobic interactions (Fig. S.6.14). Our results show the potential of this fragment from Hydroxychloroquine in the Covid-19 treatment. The non-metabolized Hydroxychloroquine interacted with Arg182 and RNA through hydrophobic interactions and with the residue Asp343 through hydrogen bond, in the RdRp binding site (Fig. 3). In the M^pro^ site, it was observed, from docking calculations, that only one type of intermolecular interaction occurred, the hydrophobic one, with the catalytic residue His41, as well as with the residues Met165 and Met49 (Fig. 4).

### EI-MS in drug metabolism and pharmacokinetics

The better comprehension of the expression and regulation of cytochrome P450 (CYP) and other enzyme systems has significantly enhanced the ability to understand the role of drug metabolism in early drug development^47^. In this line, drug metabolism and pharmacokinetics have a central role in drug discovery, assisting in designing and selecting the most promising drug candidates. This tool can lead to advanced insights on the processes that control absorption, distribution, metabolism, excretion and toxicity (ADMET) of the final compound. EI-MS is one of the key technologies applied in this kind of simulation^48^. The metabolism study allows for structural modifications during the optimization process, which were usually based on empirical methods, past experience, and even intuition^47^. However, with the advances in computational chemistry and processing capacity, these processes are far more efficient^49–58^.

Drug metabolism reactions are usually divided into two categories: Phase I and Phase II reactions. Among the reactions of Phase I, we can cite the hydrolysis of ester or amide groups to their respective acid and alcohol/amines, hydroxylation of aromatic and aliphatic carbons, heteroatom dealkylation (secondary/tertiary amines, ethers, thioethers), and heteroatom oxidation (N-, S-oxidation). Regarding Phase II, we can cite the metabolic pathways, including glucuronidation, sulfation, and glutathione conjugation^47^.

Some works dedicated to the study of by-products from metabolism processes. For instance, in the case of Chloroquine, the two most important metabolites were desethyl chloroquine and bisdesethyl chloroquine, through dealkylation in the liver. According to the authors, these metabolites have pharmacologic activity and are thought to be approximately as toxic as the non-metabolized drug^59^. Hu et al. investigated the pharmacokinetic behavior and tissue distribution of Rendesivir and its metabolites^60^, as well as it is approached in other studies^61,62^.

In this technique of metabolism prediction, the chemical identification of mass spectrometric signals is important to provide conversion of analytical data to biological knowledge about metabolic pathways. Databases can provide knowledge on thousands of endogenous and exogenous metabolite species. The technique is based on the combination of accurate mass data for a large collection of metabolites, and through the computational analysis of these species, a range of data on ADMET can be obtained, as well as reactivity and interaction modes within the molecular target^63^.

## Finals remarks

Herein, our main goal was to investigate the interaction modes of diverse drugs for the Covid-19 treatment, as well as the interaction modes of their fragments formed in the study of metabolism by using the tool QCEIMS. Our outcomes indicate that the fragments of these drugs can also target the viral M^pro^ enzyme and RdRp polymerase. It is noteworthy that each species' molecular docking pose showed that they could fit accurately within the substrate-binding pocket. Thus, the analysis of the fragments generated from each drug is a crucial step to better comprehend the action modes of these drugs toward two different important molecular targets for Covid-19 treatment, that is, M^pro^ and RdRp. In this work, we noticed that the fragments interacted with RNA of different ways, indeed the larger fragments as well as the fragments with hydrogen bond donors contribute to more stabilized interactions with RNA. This same trend can be observed for the interaction of these fragments in the M^pro^ binding site, this is because larger fragments more often interact with both residues of the catalytic dyad, Cys145 and His41. Among the compounds analyzed, Nitazoxanide was the one that provided a more stable receptor-ligand complex (− 5.6 kcal mol^−1^) within M^pro^ binding site. The amount of energy required for a molecule to bind to a specific molecular target interferes with its biological activity, because the more stable the complex formed, the less energy is required for this interaction to occur. Therefore, when comparing the results obtained for all analyzed ligands, it is clear that the ligands that presented binding energies lower than that of the natural product, that is, those that presented lower energy values, in theory, are more active, as it will bind more easily to the molecular target. Thus, it is expected that Nitazoxanide presents good results of biological activity when performed experimental studies. In the RdRp binding site, Remdesivir presented the lowest interaction energy (− 9.9 kcal mol^−1^), that is, this drug is more stabilized in the target site than the other drugs investigated.

According to the metabolism study of the drugs approached in this work, these drugs can generate more stabilizing or less stabilizing fragments, even with fragments interacting better in the target site than the non-metabolized drug. These trends can vary according to the drug investigated, as shown along of this theoretical study. Equally important is the formation of more toxic or less toxic fragments. Some drugs, for example, revealed fragments less toxic than the drugs themselves. The set of the analyses developed in this study can bring about great contributions for the development of drugs for Covid-19 treatment, as well as for the development of drugs for the treatment of other diseases.

## Supplementary Information

Supplementary Information.
