# Supplementary material for: Effect of drug metabolism in the treatment of SARS-CoV-2 from an entirely computational perspective
Source: Sci Rep. 2021 Oct 7;11:19998. doi: 10.1038/s41598-021-99451-1 (PMC8497625; doi:10.1038/s41598-021-99451-1)
Supplement: Supplementary file 1 — Supplementary Information. [file 41598_2021_99451_MOESM1_ESM.pdf]

SUPPLEMENTARY MATERIAL

EFFECT OF DRUG METABOLISM IN THE TREATMENT OF SARS-COV-2 FROM AN ENTIRELY COMPUTATIONAL PERSPECTIVE

João Paulo Almirão de Jesus,[1] Letícia Cristina Assis,[2] Alexandre Alves de Castro,[2] Elaine Fontes Ferreira da Cunha,[2] Eugenie Nepovimova, Kamil Kuca, Teodorico de Castro Ramalho[2] and Felipe de Almeida La Porta[1*]

[1]Laboratory of Nanotechnology and Computational Chemistry, Federal Technological University of Paraná, Avenida dos Pioneiros 3131, CEP 86036-370, Londrina, Paraná, Brazil.

[2]Department of Chemistry, Federal University of Lavras, CEP 37200-000, Lavras, Minas Gerais, Brazil.

[3] Department of Chemistry, Faculty of Science, University of Hradec Kralove, Rokitanskeho 62, 500 03 Hradec Králové, Czech Republic

Corresponding author

E-mail: kamil.kuca@uhk.cz; felipe_laporta@yahoo.com.br or felipelaporta@utfpr.edu.br.

SUMMARY

S.1 – CARTESIAN COORDINATES OF THE OPTIMIZED STRUCTURES..3

S.2 – OPTIMIZED STRUCTURES, BOND LENGTHS OF FUNCTIONAL GROUPS AND ESP MAPS (WITH SURFACE ISOVALUE OF 0.0004) OF a) FAVIPIRAVIR, b) GALIDESIVIR; c) NITAZOXANIDE, d) REMDESIVIR, AND e) RIBAVIRIN, GENERATED IN THE GAUSSVIEW 6.0 HTTPS://GAUSSIAN.COM/GAUSSVIEW6/..11

S.3 – ESTIMATED a) UV-VIS AND b) ECD SPECTRA OF THE DRUGS...12

S.4 - ELECTRONIC PROPERTIES OF THE DRUGS...13

S.5 - THEORETICAL (BLACK) AND EXPERIMENTAL (PINK) EI-MS SPECTRA OF a) CHLOROQUINE AND b) HYDROXYCHLOROQUINE, AND THEIR RESPECTIVE TRAJECTORIES, BY MEANS OF GFN2-XTB. THEORETICAL (BLACK) AND EXPERIMENTAL (PINK) EI-MS SPECTRA OF c) CHLOROQUINE AND d) HYDROXYCHLOROQUINE RESULTED FROM THE GFN1-XTB METHOD. STRUCTURES. PLOTS GENERATED IN THE GRACE SOFTWARE HTTPS://PLASMA-GATE.WEIZMANN.AC.IL/GRACE/ 14

S.6 – PHARMACOPHORIC MAPS SHOWING THE INTERMOLECULAR INTERACTIONS OF THE FRAGMENTS OF DIVERSE DRUGS IN THE RDRP AND MPRO ACTIVE SITE…………..17

REFERENCES..32

S.1 – CARTESIAN COORDINATES OF THE OPTIMIZED STRUCTURES

FAVIPIRAVIR

0 1

C	2.18836100	0.64798800	0.04520100
C	1.75736500	-0.64557300	0.03915200
C	-0.47168600	-0.06847900	-0.00944400
C	-0.16571900	1.38067200	-0.08102400
H	3.22924300	0.94356700	0.08491800
F	2.65106500	-1.65708700	0.06968800
N	0.46588900	-0.98812200	0.01742300
N	1.22782000	1.61227600	-0.00278400
H	1.48788200	2.59244500	-0.02767800
O	-0.93389800	2.31765400	-0.21524400
C	-1.90803200	-0.54426200	0.05786800
O	-2.81863700	0.18619200	0.42024500
N	-2.07818700	-1.84818500	-0.30794200
H	-1.27791300	-2.45166800	-0.42699400
H	-2.98890700	-2.25517700	-0.15485400

GALIDESIVIR

0 1

C	2.77059800	0.52044300	-0.28610200
C	1.69928000	-0.11565800	0.37586900
C	3.16146700	-1.30239800	1.60272600
C	4.08024200	0.14410400	0.05498500
C	0.85416700	1.36213900	-1.09324400
C	0.47933500	0.42573600	-0.15619600
H	3.36463100	-2.03576900	2.37904400
H	2.73809400	2.12777700	-1.69522000
H	0.23295400	2.00616700	-1.69820100
N	5.20536800	0.70855000	-0.51499200
H	6.06219000	0.22547300	-0.27442400
H	5.14863200	0.98452800	-1.48558500
N	4.25409900	-0.76785700	1.01620100
N	1.88509800	-1.05001400	1.33821900
N	2.23358300	1.41339200	-1.19494300
C	-0.90411800	0.02489600	0.24848500
C	-1.43130600	-1.29696200	-0.34581400
H	-0.91169600	-0.09945000	1.34541600
C	-3.25299000	0.30899400	-0.05223500
C	-2.93650800	-1.21873500	0.00516900
H	-0.93749600	-2.17977000	0.06319100
H	-3.88728100	0.50508100	-0.92783600
H	-3.09280800	-1.59205700	1.02484500
N	-1.93702800	0.96008200	-0.23739200
H	-1.90273300	1.86818400	0.22002700
C	-3.98967900	0.79293900	1.19928500
H	-4.94565600	0.25905900	1.29886900
H	-3.39349600	0.58636300	2.09496800
O	-4.18723300	2.21007100	1.20027400
H	-4.84949900	2.43462700	0.53302700

O	-3.74305100	-2.01203200	-0.84196900
H	-3.26805800	-2.06964000	-1.68862100
O	-1.28112400	-1.35961200	-1.76533500
H	-1.33728600	-0.45005200	-2.10047900

NITAZOXANIDE

0 1

C	4.94095400	-1.43761600	0.26540500
C	4.12481100	-2.55520600	0.06720800
C	2.74670000	-2.39477700	-0.02006400
C	2.14839300	-1.12581800	0.08509300
C	2.99426700	-0.01599700	0.27478700
C	4.37582200	-0.16788400	0.37066500
H	6.01826800	-1.54995300	0.33812100
H	4.56095000	-3.54535600	-0.01550200
H	2.09196300	-3.24564100	-0.17135900
H	4.99904300	0.70617200	0.51557500
O	2.43007000	1.25665700	0.43548800
C	2.80946300	2.30299400	-0.40541700
C	2.08057800	3.55921800	-0.01626700
H	2.40117400	4.37312700	-0.66551900
H	0.99875000	3.41711800	-0.10910800
H	2.29009100	3.80680000	1.02876200
O	3.60362500	2.17060200	-1.29784300
C	0.65371400	-1.10703100	-0.01741000
O	0.00924400	-2.09445900	-0.36144000
N	-0.00151900	0.06406000	0.31379700
C	-1.36281600	0.24553100	0.26555900
C	-3.23126100	1.38910700	0.46496800
C	-3.74677500	0.17994100	0.07329700
H	-3.82286300	2.26954600	0.67929900
S	-2.49013900	-1.01301400	-0.18631700
N	-1.87519800	1.42395300	0.57305800
N	-5.12376300	-0.13567200	-0.12273000
O	-5.95440200	0.75749200	0.07846600
O	-5.39330100	-1.28940700	-0.48258100
H	0.54323500	0.86817000	0.60825800

REMDESIVIR

0 1

P	-1.28189400	1.67359000	-0.61719300
O	-0.11630400	2.23915900	-1.34205700
O	1.17063900	2.86871300	1.83719900
H	1.38645300	2.87255500	2.78296500
O	2.38137200	-0.52716500	1.58182500
O	3.24784300	1.89344400	3.15177300
H	4.09604900	1.69329300	3.57383500
N	4.55130200	-1.21636800	-0.47081800
N	-2.38671900	0.98027300	-1.66885200
H	-1.93206800	0.61947000	-2.50301100
O	-0.94010900	0.60559800	0.55179100
O	-3.89799900	-1.93036500	-0.13005000
N	5.37363000	-0.61341100	3.23290400
O	-2.08143100	-1.81587600	-1.47589000
N	5.80040700	-2.32061600	-3.70819100
H	5.97624000	-3.22024100	-4.13107900
H	5.64416600	-1.52934700	-4.31102700
N	4.54887600	-2.37858700	0.23966800
C	-3.49587200	0.15007400	-1.20802800
H	-3.86155000	0.55709200	-0.26110700
O	-2.19304100	2.65244000	0.29698700
N	5.37733900	-3.45640700	-1.74674800
C	4.59602800	-0.35384600	2.41008600
C	3.63203700	0.16709900	1.41418800
C	1.86405600	1.79289700	1.24542900
H	1.84942500	1.97929800	0.16925200
C	3.31262300	1.65729700	1.75556100
H	4.01488600	2.34609800	1.27651900
C	-3.05117300	-1.29754200	-0.95816400
C	4.33362300	0.92558300	-1.03087000

H	4.11284800	1.98190700	-0.98325600
C	5.37275400	-2.31334800	-2.41961000
C	4.17031400	0.00732900	0.01191500
C	4.95397100	-1.09612800	-1.80077500
C	0.28979300	-0.15060900	0.51470100
H	0.73941000	-0.12209400	-0.48168200
H	0.03230500	-1.18414100	0.75440200
C	4.96896000	-3.41003300	-0.45170000
H	4.98195800	-4.34833500	0.09343600
C	-3.81070700	5.80984400	-0.66754700
H	-4.77492200	6.15603900	-1.02823200
C	1.27064600	0.39516600	1.55254400
H	0.79611400	0.40806300	2.54151800
C	-2.78321600	6.72381000	-0.41882900
H	-2.94568900	7.78435700	-0.58621200
C	-4.64187400	0.17041700	-2.23500300
H	-4.97610400	1.19957100	-2.38788800
H	-5.48567400	-0.43209100	-1.88805900
H	-4.30338000	-0.22732200	-3.19800000
C	-3.61542700	-3.32856000	0.15731200
H	-2.61402300	-3.38571900	0.59017900
H	-3.60304800	-3.88069700	-0.78755000
C	4.82127000	0.24552100	-2.16074200
H	5.05002500	0.68379600	-3.12165600
C	-4.68300400	-3.84777100	1.11874400
H	-4.66287200	-3.19089300	2.00064000
C	-2.36436700	4.01202500	0.01170900
C	-3.60458700	4.44409400	-0.45385500
H	-4.39059900	3.71941300	-0.63762900
C	-1.54701400	6.26866900	0.05076400
H	-0.74679700	6.97564700	0.24951300
C	-1.32771700	4.90730800	0.27430700

H	-0.38072300	4.53383300	0.64912300
C	-6.11094200	-3.75940300	0.53060100
H	-6.81532500	-4.03198400	1.32732900
H	-6.32101100	-2.71230200	0.28407200
C	-4.32823100	-5.27495700	1.59538900
H	-4.14345500	-5.92321200	0.72848400
H	-5.21154700	-5.68976000	2.09789700
C	-6.38985300	-4.63372100	-0.70065700
H	-7.42454300	-4.50056500	-1.03398800
H	-6.25006900	-5.69892600	-0.48855100
H	-5.74293700	-4.37497100	-1.54631600
C	-3.13357200	-5.35574500	2.55654900
H	-2.19695900	-5.03709800	2.08730100
H	-2.98617100	-6.38337300	2.90506400
H	-3.29470600	-4.72579800	3.43912100

RIBAVIRIN

0 1

C	2.58967500	0.40027500	0.40092300
O	1.45090600	0.60481800	1.25984100
C	0.52229000	-0.45689700	1.09699500
C	1.11314400	-1.44320600	0.07592500
C	2.07881000	-0.53730800	-0.70359200
H	3.38166800	-0.11097100	0.96544400
H	0.34267100	-0.95167900	2.05555800
H	0.33423200	-1.89159100	-0.54852200
H	1.51630200	0.03942900	-1.45282600
O	1.82185500	-2.42734600	0.81027700
H	2.50523200	-2.78789300	0.22407900
O	3.08406200	-1.31736400	-1.32130800
H	3.74987600	-0.70671000	-1.67338200
C	3.10173700	1.73880800	-0.11677900
H	2.31265500	2.25524700	-0.67285600
H	3.42231200	2.38754100	0.70588800
O	4.17270800	1.49264200	-1.04235000
H	5.01968100	1.56047600	-0.58456400
N	-0.74598100	0.13217400	0.63245400
C	-1.12974800	1.43301500	0.60256500
H	-0.47573600	2.24740100	0.87124100
C	-2.74268100	0.20951100	0.01634000
N	-1.77501200	-0.66448600	0.27029500
N	-2.38900300	1.51703600	0.20761700
C	-4.10725200	-0.20554600	-0.44581200
O	-4.41766300	-1.37393200	-0.63708800
N	-4.95092700	0.84916400	-0.62677700
H	-5.89307700	0.66498100	-0.93531600
H	-4.64014500	1.79408400	-0.45423600

S.2 – OPTIMIZED STRUCTURES, BOND LENGTHS OF FUNCTIONAL GROUPS AND ESP MAPS (WITH SURFACE ISOVALUE OF 0.0004) OF a) FAVIPIRAVIR, b) GALIDESIVIR; c) NITAZOXANIDE, d) REMDESIVIR, AND e) RIBAVIRIN, GENERATED IN THE GAUSSVIEW 6.0 [HTTPS://GAUSSIAN.COM/GAUSSVIEW6/](https://gaussian.com/gaussview6/)

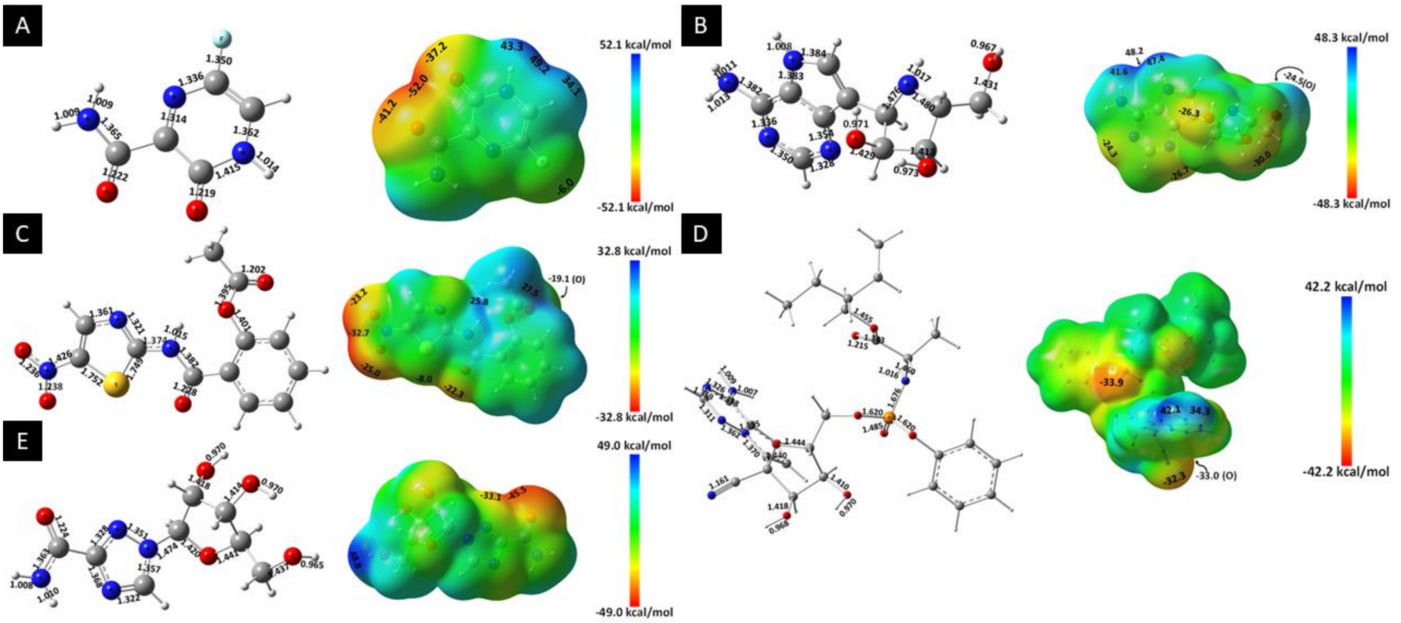

In the figure above, it is illustrated the bond lengths of the functional groups that compose the drugs' structures and their ESP maps. In general, the electronic density is distributed in a polar manner for all the molecules, in which the red areas show higher negative potential (mostly due to electron acceptor oxygen-derivate groups such as nitro or hydroxide) and the blue areas positive potential (mainly due to the electron-donor nitrogen atoms). In this framework, the Favipiravir polar structure shows two regions suitable for interactions with external molecules between the carbonyl groups and the neighbor amine. For the Galidesivir drug, the charge is distributed moderately on the hydroxide groups and heavily on the outermost amines, also it is worth notice that amine groups in the aromatic rings show electron-acceptor character. The Nitazoxanide molecule is very polarized with many regions with positive potential, showing the higher positive charge next to the ester group, and heavily negatively charged in the nitro region. Due to the large molecular structure, the charge distribution of Remdesivir is slightly more homogenous than the other drugs, having polar character in the region containing the most functional groups, thus presenting a portion with high positive potential that is close to an electron-acceptor area due to the hydroxide group. It is also noticed that for the Remdesivir molecule, the sulfur center shows slightly intense negative potential because of the oxygen-derivate groups. Lastly for the Ribavirin drug, the charge is distributed mostly homogenously around the structure body and shows two poles with high negative and positive potentials, due to a hydroxide and amine groups, respectively.

S.3 – ESTIMATED a) UV-VIS AND b) ECD SPECTRA OF THE DRUGS

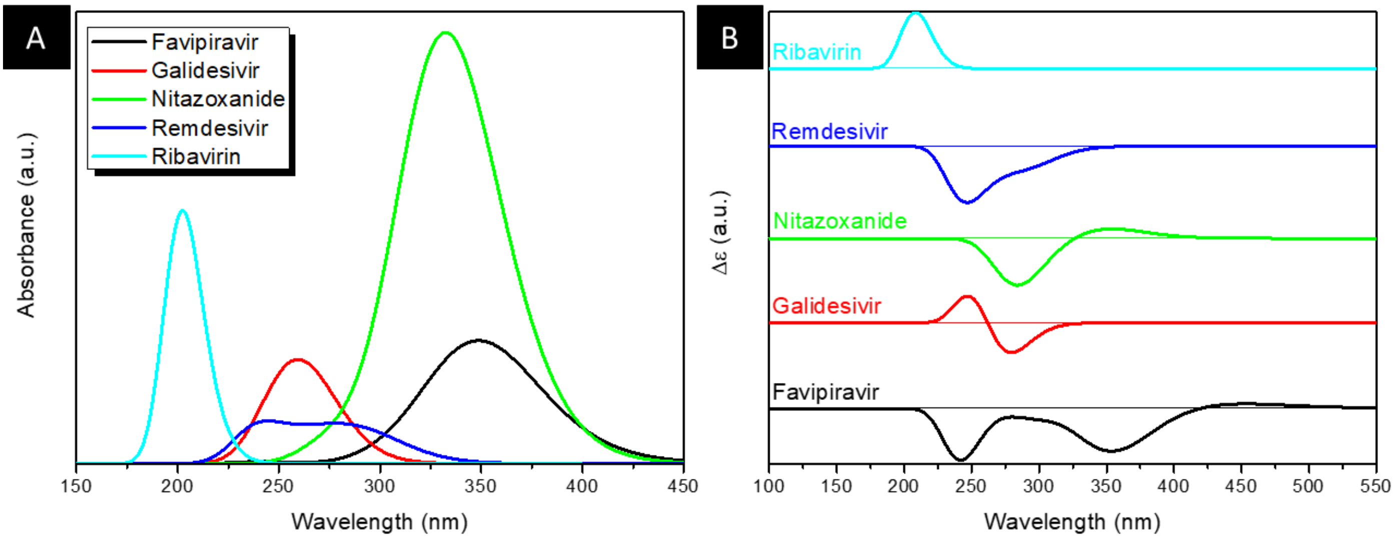

In the Figure S.3.A are shown the UV-Vis spectra of the drugs. The Nitazoxanide molecule shows the most intense absorbance at 333 nm (HOMO → LUMO), followed by the slightly minor absorbance of Ribavirin at 202 nm (HOMO-2 → LUMO), then moderate intensities for Favipiravir and Galidesivir, respectively at 350 nm (HOMO → LUMO) and 257 nm (HOMO-3 → LUMO), and lastly the low intensity peaks of Remdesivir at 241 nm (HOMO → LUMO+2) and 269 nm (HOMO → LUMO+1). From the ECD spectra shown in S.3.B, it is observed that Favipiravir drug shows two negative signals at 241 nm and 350 nm, one positive signal at 247 nm and one negative at 269 nm for the Galidesivir, the inverse for Nitazoxanide with one negative at 287 nm and positive around 340 nm, a broad positive signal in the range of 215-350 nm for Remdesivir and a single positive peak at 209 nm for Ribavirin. Information regarding the electronic properties of the studied molecules are found in S.4.

S.4 - ELECTRONIC PROPERTIES OF THE DRUGS.

Molecule	LUMO - HOMO (eV)	Hardness (eV)	Softness (eV)	Mulliken Electronegativity (eV)	Electrophilicity (eV)
Favipiravir	3.87	1.94	0.52	-5.02	50.40
Galidesivir	5.21	2.60	0.38	-3.69	27.24
Nitazoxanide	4.09	2.05	0.49	-5.12	52.54
Remdesivir	4.82	2.41	0.41	-3.92	30.74
Ribavirin	6.02	3.01	0.33	-4.14	34.21

The Table above organizes the electronic properties of the drugs, in which are in agreement with the expected values for molecules. It is observed that the Favipiravir shows the smallest LUMO-HOMO energy, followed by Nitazoxanide, Remdesivir, Galidesivir and then Ribavirin, which these energies may in principle indicate the order of reactivity of these molecules, being Favipiravir the most reactive and Ribavirin the lowest. As expected, the hardness follows the same tendency and is inverse to the softness, indicating that the calculations are in order. As for the electronegativity, it increases from Galidesivir to Remdesivir, Ribavirin, Favipiravir and Nitazoxanide, which is the same tendency as the electrophilicity.

S.5 - THEORETICAL (BLACK) AND EXPERIMENTAL (PINK) EI-MS SPECTRA OF a) CHLOROQUINE AND b) HYDROXYCHLOROQUINE, AND THEIR RESPECTIVE TRAJECTORIES, BY MEANS OF GFN2-XTB. THEORETICAL (BLACK) AND EXPERIMENTAL (PINK) EI-MS SPECTRA OF c) CHLOROQUINE AND d) HYDROXYCHLOROQUINE RESULTED FROM THE GFN1-XTB METHOD. STRUCTURES. PLOTS GENERATED IN THE GRACE SOFTWARE [HTTPS://PLASMA-GATE.WEIZMANN.AC.IL/GRACE/](https://plasma-gate.weizmann.ac.il/grace/)

A

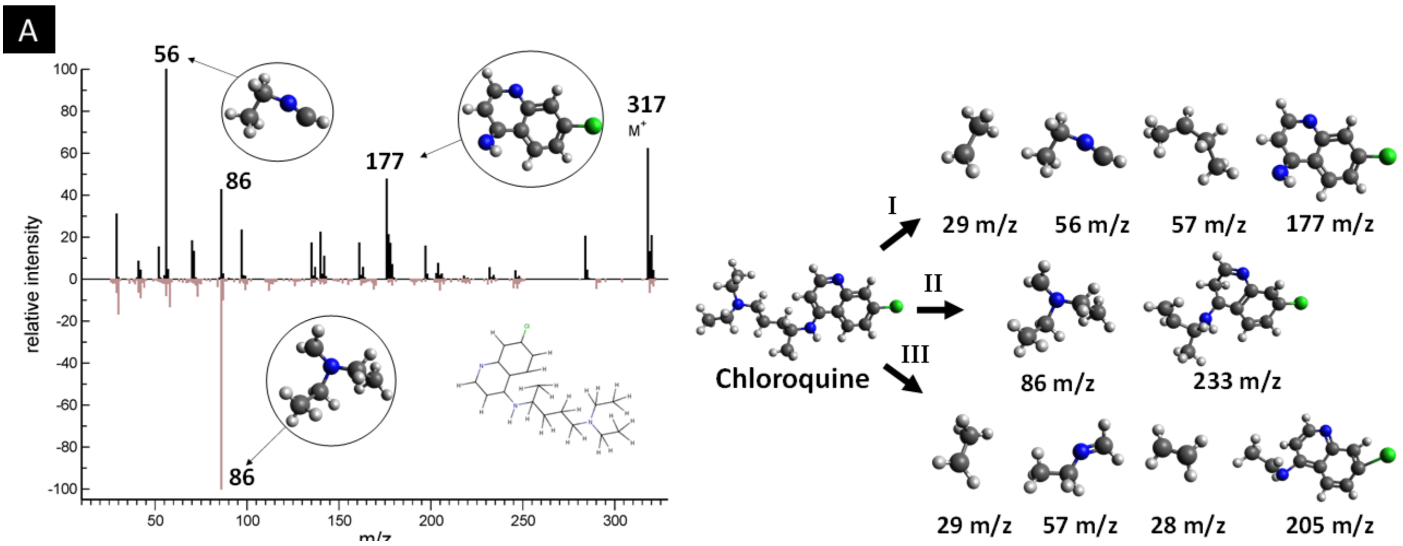

B

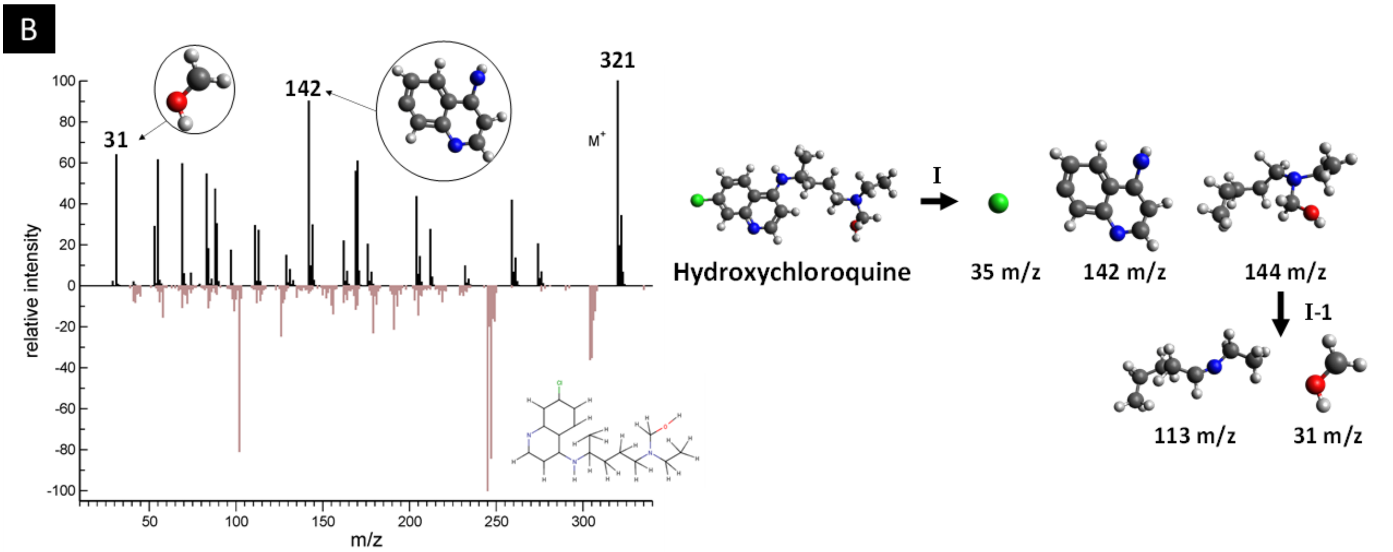

C

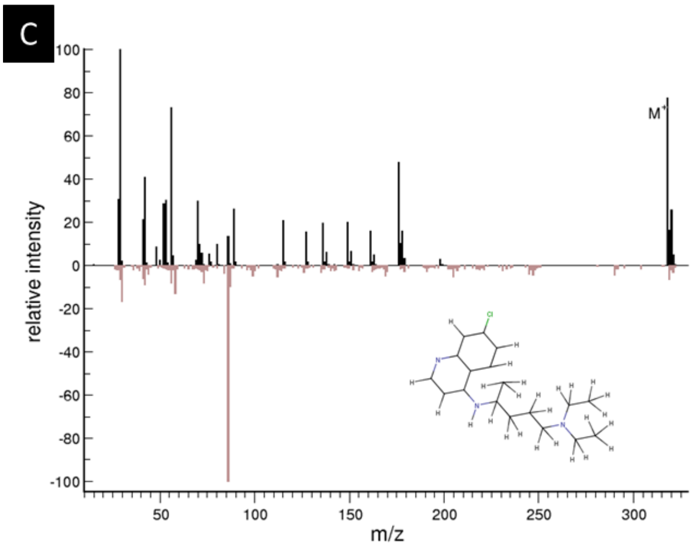

D

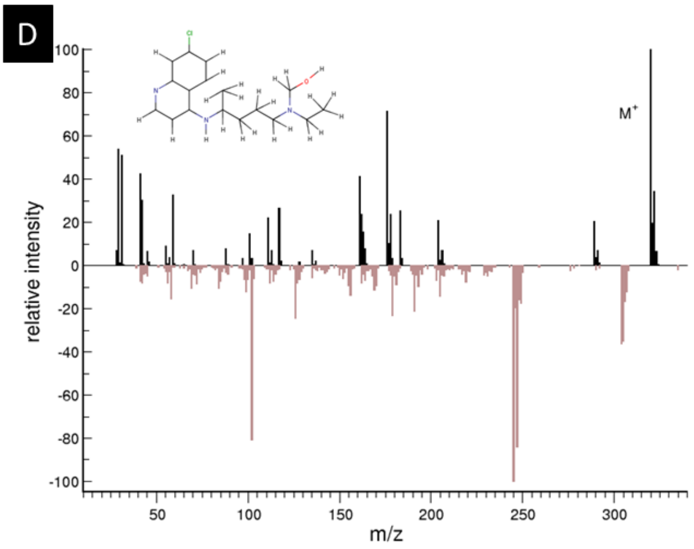

As seen in the S.5.A above, the EI-MS diagram from the GFN2-xTB method, in principle, presented most of the intermediaries of the Chloroquine molecule, in agreement with the experimental data profile (NIST MS 42361), with slight deviation of intensity. The most intense signal 86 m/z and its respective intermediary are also identified in the theoretical plot and the resultant trajectory described. As for the Hydroxychloroquine spectra in S.5.B, the most intense peaks from the experimental data (NIST MS 246973) are not identified in the theoretical data, still, some of the signals are found with distinct intensity from the experimental spectra. In order to evaluate the results between the theoretical methods, S.5.C shows the EI-MS spectra of the Chloroquine obtained using the GFN1-xTB method in comparison with the same experimental profile. In this manner, the spectra from the GFN1-xTB did not match the intermediaries mass/charge rate and intensity as well as its analog method, reveling that the GFN2-xTB is the best option for this calculation. Transitioning to the Hydroxychloroquine in S.5.D, the spectra acquired from the GFN1-xTB approach did not show significant improvements over the GFN2-xTB, and the most intense signals of the NIST profile are not identified as well. In general, an increasing on the molecular dynamics parameters could lead to a better prediction of the EI-MS spectra and its intermediaries in exchange of meaningful computational cost, however, as the current methodology with GFN2-xTB provided satisfactory results for the Chloroquine drug, it has been chosen as the default semiempirical method to the study of the other drugs. For more information regarding the characterization and details of Chloroquine and Hydroxychloroquine, please check our other works with these drugs.[1,2]

Henceforth, the discussion of the EI-MS spectra and trajectories will be done in the context of xenobiotics metabolism, evaluating the obtained intermediaries as drug by-products, their metabolism and toxicity when possible. Thus, returning to the Chloroquine drug, the spectra and trajectories are shown in S.5.A. The Chloroquine molecule contains polar amine and chloride groups in its structure, showing an aromatic region with more polar character than the other extremity. The first trajectory showed the fragmentation of Chloroquine around the amine that bond the aromatic and the alkane regions, leading to the following intermediaries: the I-177 m/z 7-chloro-4-aminoquinoline, containing the aromatic region, deprotonated amine and chloride polar groups, is a toxic and major metabolite from the oxidation of Chloroquine by the cytochrome P-450 enzyme;[3,4] the deprotonated I-57 m/z butane and I-29 m/z ethane, both nonpolar hydrocarbons which can be oxidized into polar species in Phase I of metabolism; and the I-56 m/z deprotonated amine, a polar and likely water-soluble molecule that may metabolize directly in Phase II. The trajectory II leads to the high molecular mass fragment II-233 m/z similar to the I-177 m/z, with an alkane extremity that may be target of oxidation in Phase I; and the II-86 m/z, the specie also identified in the experimental spectra, show very low polar character and may be almost insoluble in water, possible target of oxidative reactions in Phase I metabolism before conjugation in Phase II. The last trajectory for Chloroquine gives the following intermediaries: the III-205 m/z, a specie like the I-177 m/z and II-233 m/z, with a shorter alkane segment which may be oxidized in Phase I, and share the behavior of its analog molecules; the

deprotonated organic molecules III-29 m/z ethane and III-28 m/z ethene, both nonpolar and likely targets to oxidizing reactions in Phase I, leading to polar conjugates to metabolize in Phase II; and the protonated form of I-56 m/z.

The S.5.B shows the EI-MS spectra and unique trajectory of the Hydroxychloroquine drug. The molecular structure of this drug is a more polar analog of the Chloroquine due to the addition of a hydroxide group. The calculations for the Hydroxychloroquine resulted in a single trajectory: the I-142 m/z, a deprotonated aminoquinoline similar to the 7-chloro-4-aminoquinoline from the metabolization of Quinoline, which is a metabolite from the Hydroxychloroquine;[5] the I-35 m/z chloride ion; and the I-144 m/z, with polar amine and alcohol groups, and the nonpolar extremities likely submitted to oxidative reactions in Phase I that may lead to smaller and polar fragments. This last fragment is further cleaved into two more species: the I-1-31 m/z molecule, which is deprotonated into a highly water-soluble and toxic formaldehyde form, being rapidly metabolized into formate by the alcohol dehydrogenase enzyme;[6,7] and the I-1-113 m/z, an amine with pentane and ethane extremities, and a possible target for Phase I oxidative reactions that have as products smaller and polar molecules, further being transformed into metabolites in Phase II.

S.6 – Pharmacophoric maps showing the intermolecular interactions of the fragments of diverse drugs in the RdRp and M^pro active site

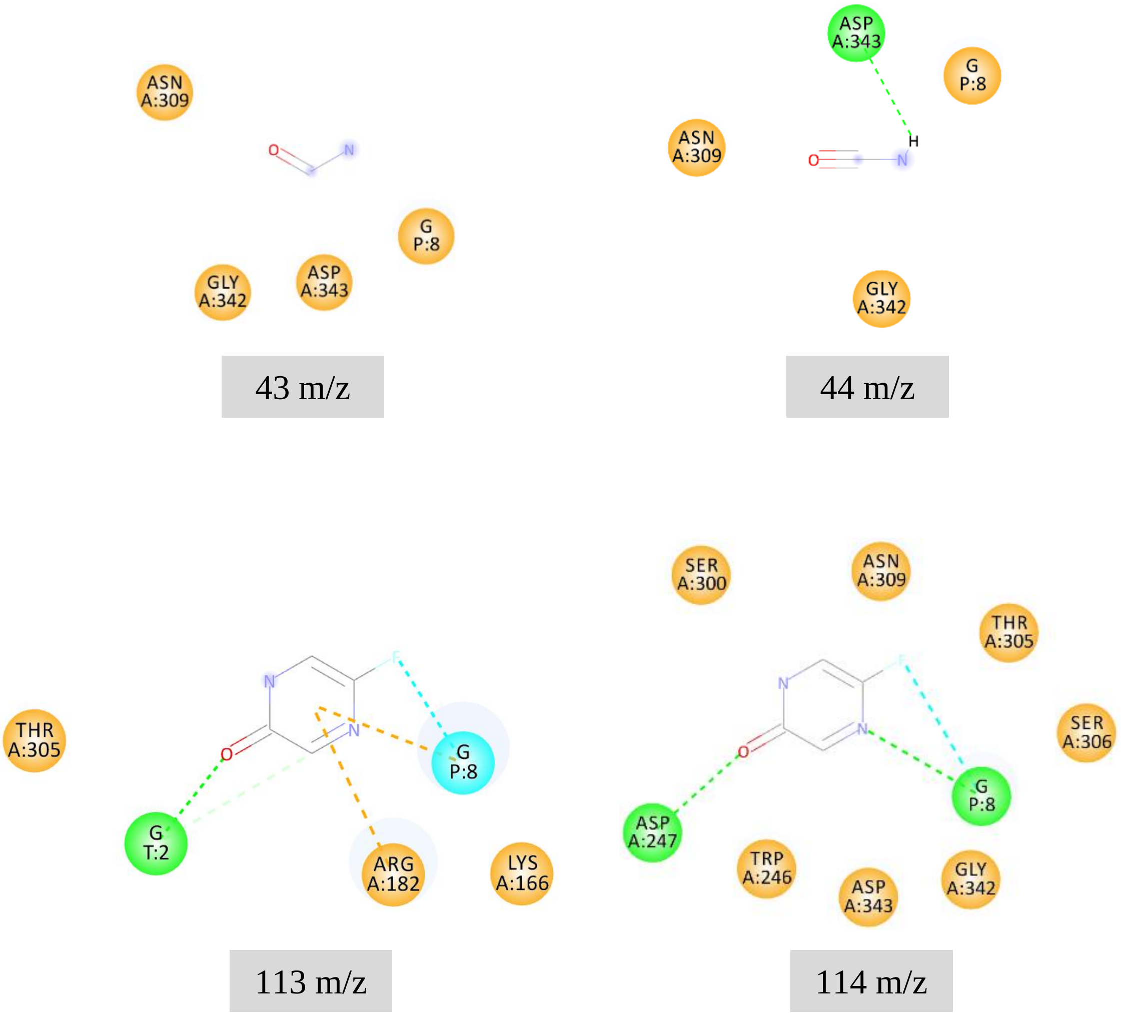

Figure S.6.1. Intermolecular interactions of the fragments of Favipiravir in the RdRp binding site. Green = hydrogen bond, Orange = hydrophobic interaction, Blue = halogen bond.

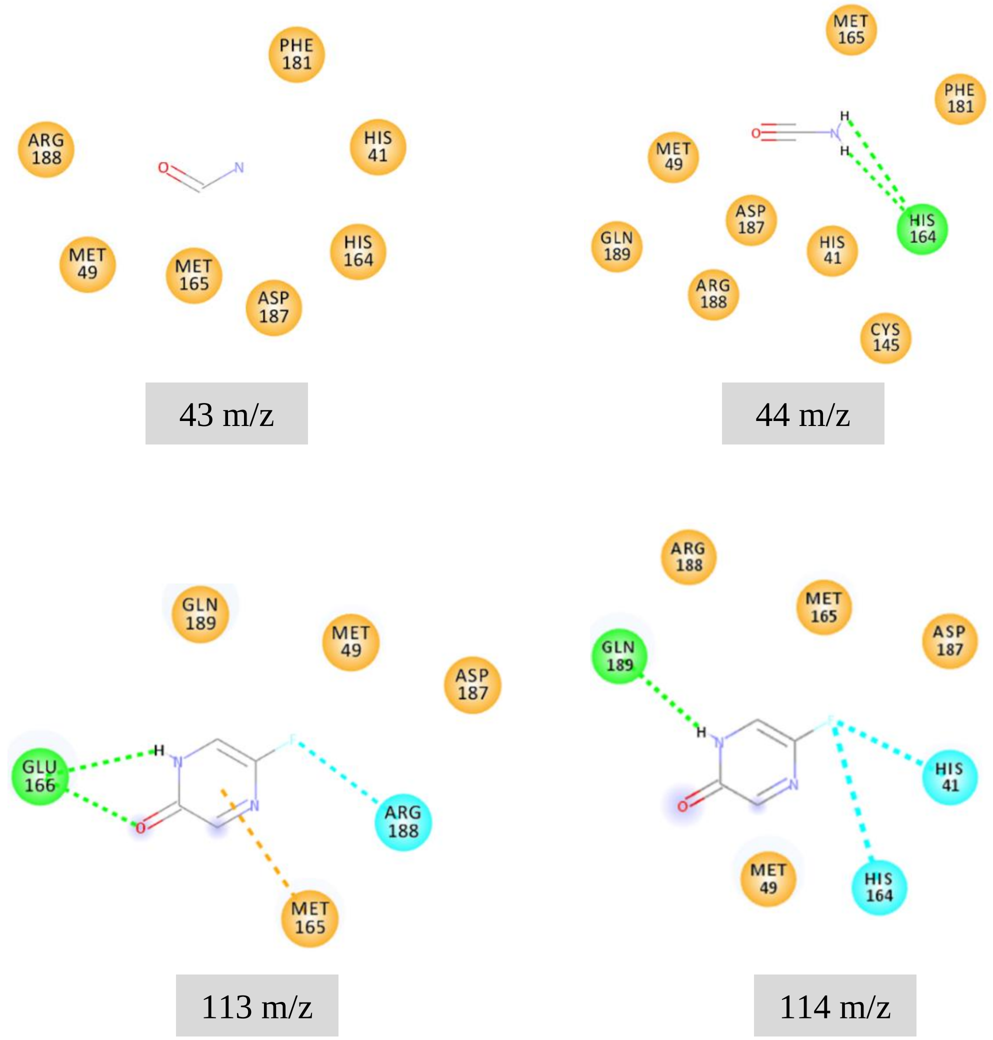

Figure S.6.2. Intermolecular interactions of the fragments of Favipiravir in the M^{pro} binding site. Green = hydrogen bond, Orange = hydrophobic interaction, Blue = halogen bond.

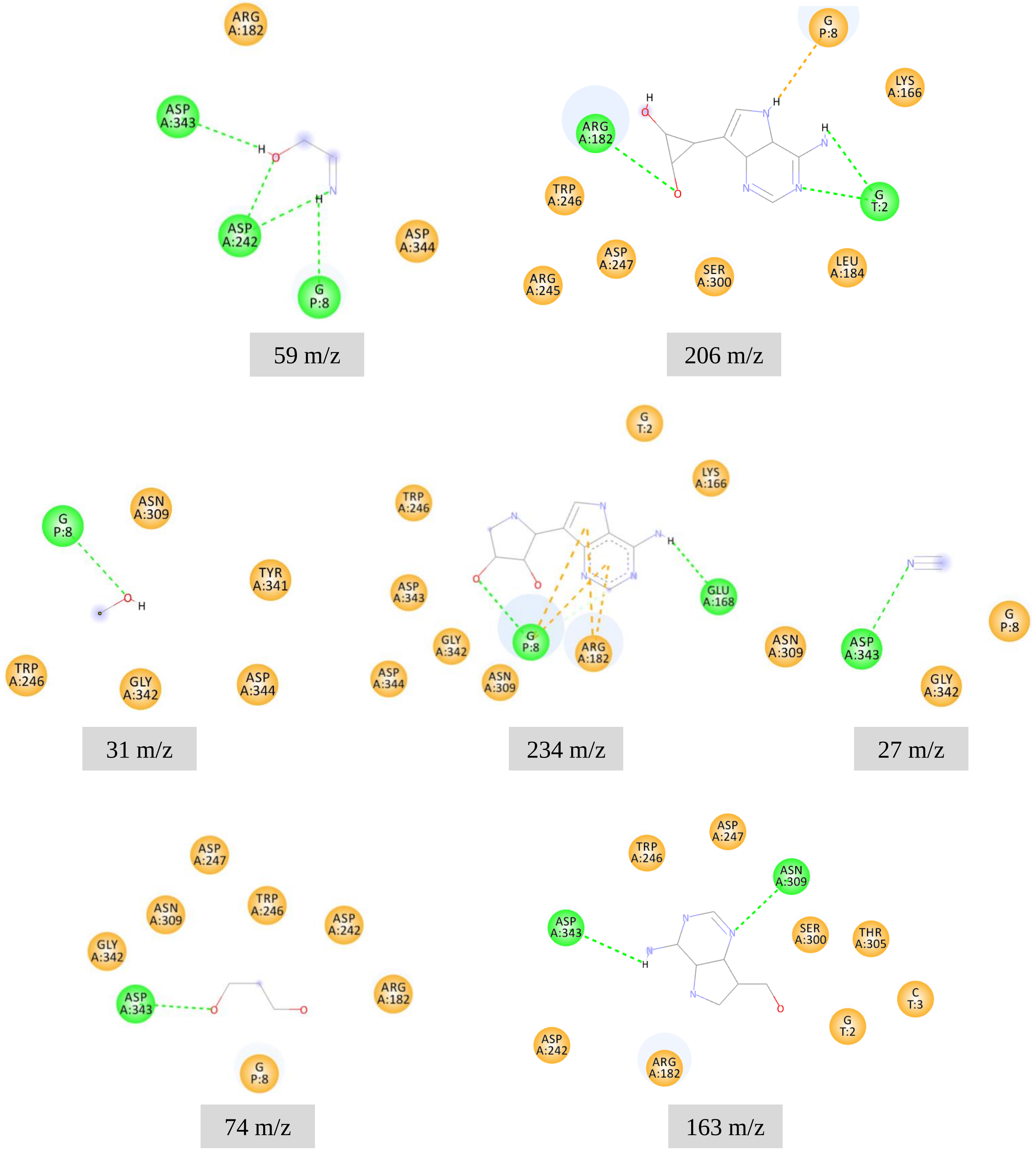

Figure S.6.3. Intermolecular interactions of the fragments of Galidesivir in the RdRp binding site. Green = hydrogen bond, Orange = hydrophobic interaction.

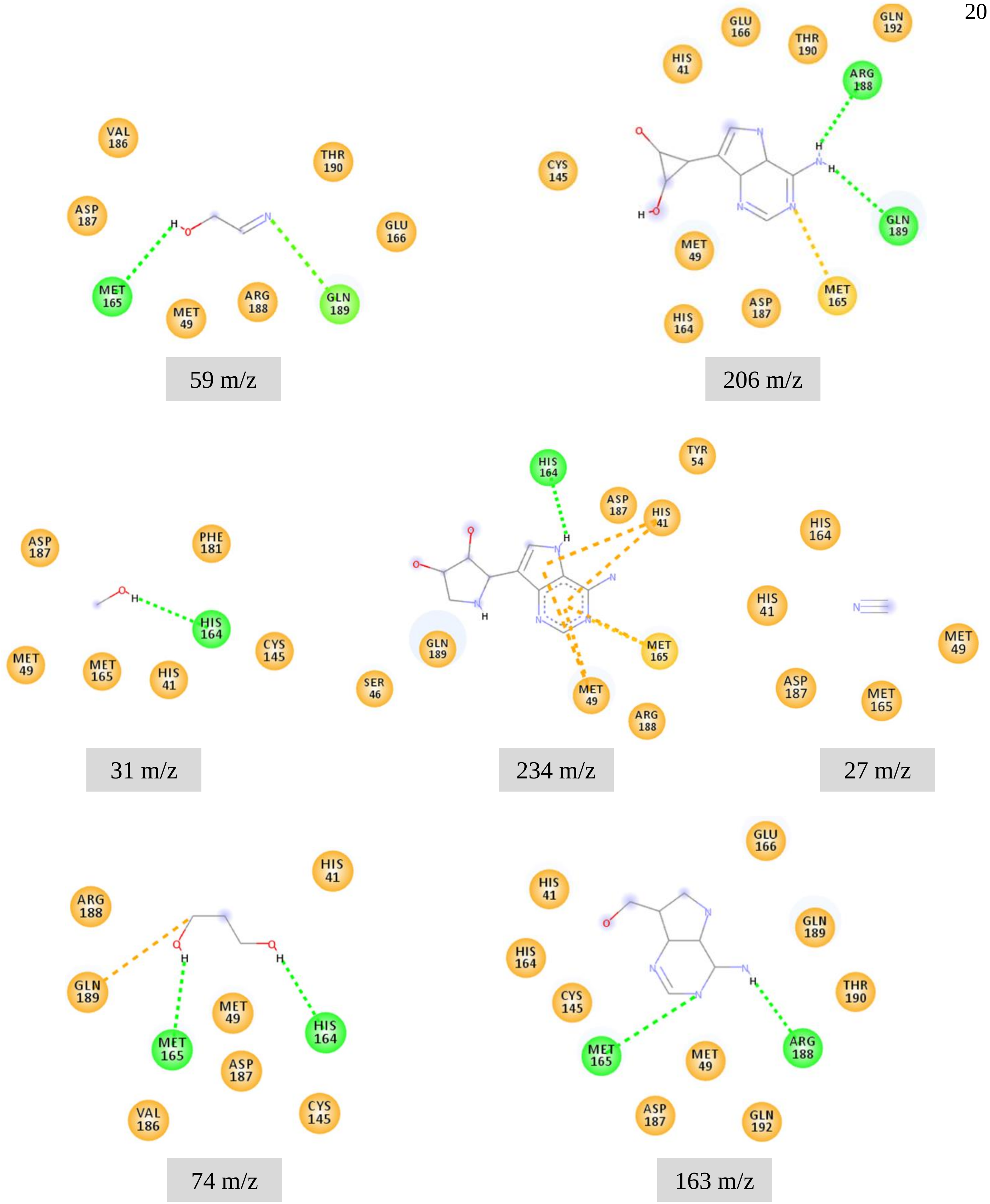

Figure S.6.4. Intermolecular interactions of the fragments of Galidesivir in the M^{pro} binding site. Green = hydrogen bond, Orange = hydrophobic interaction.

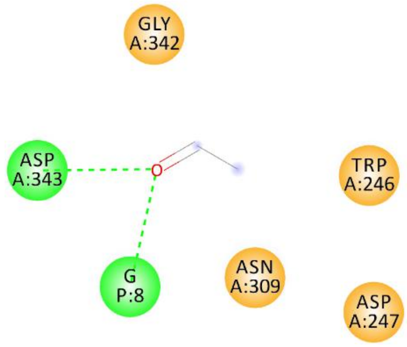

43 m/z

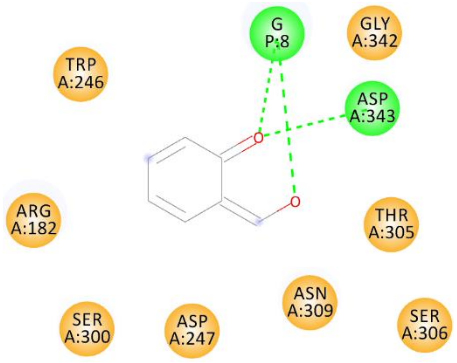

120 m/z

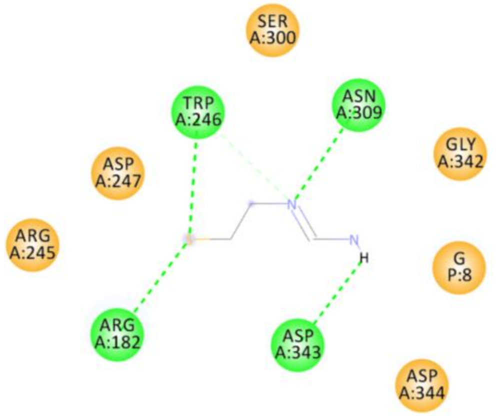

98 m/z

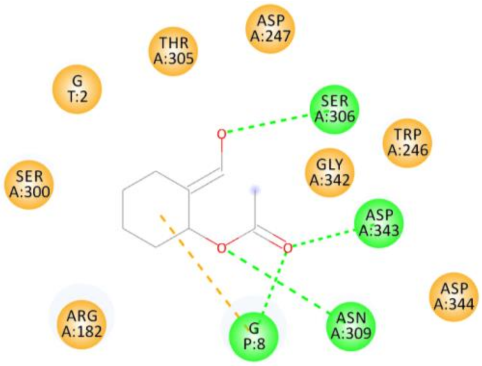

163 m/z

Figure S.6.5. Intermolecular interactions of the fragments of Nitazoxanide in the RdRp binding site. Green = hydrogen bond, Orange = hydrophobic interaction. No interaction found for fragment 46 m/z.

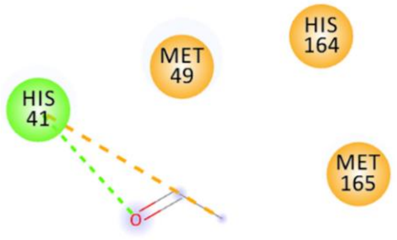

43 m/z

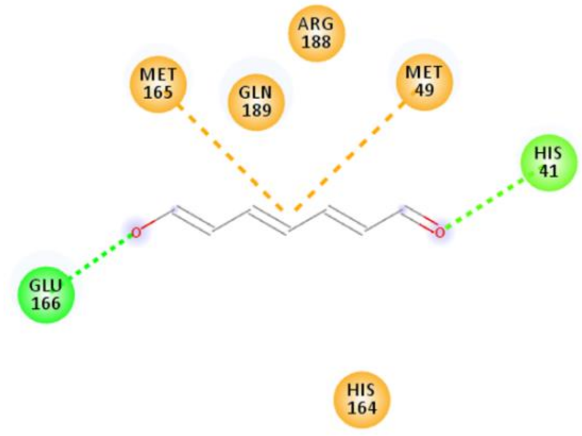

120 m/z

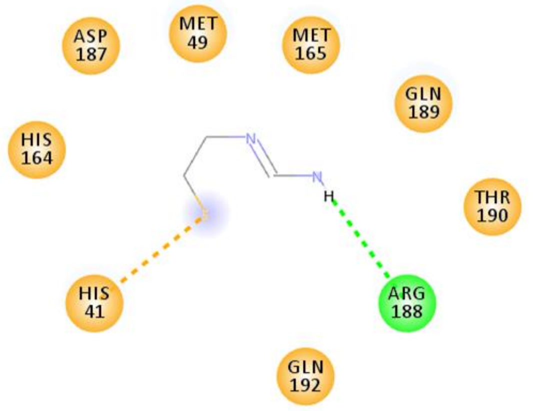

98 m/z

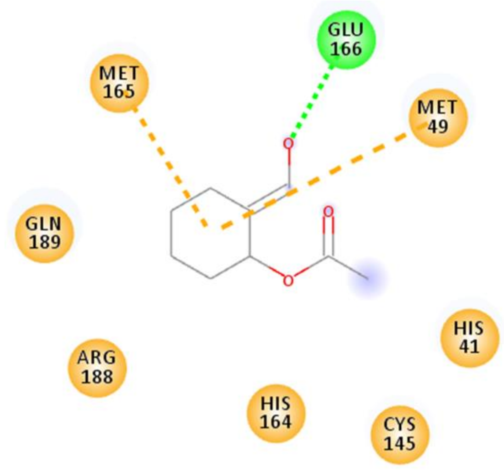

163 m/z

Figure S.6.6. Intermolecular interactions of the fragments of Nitazoxanide in the M^{pro} binding site. Green = hydrogen bond, Orange = hydrophobic interaction. No interaction found for fragment 46 m/z.

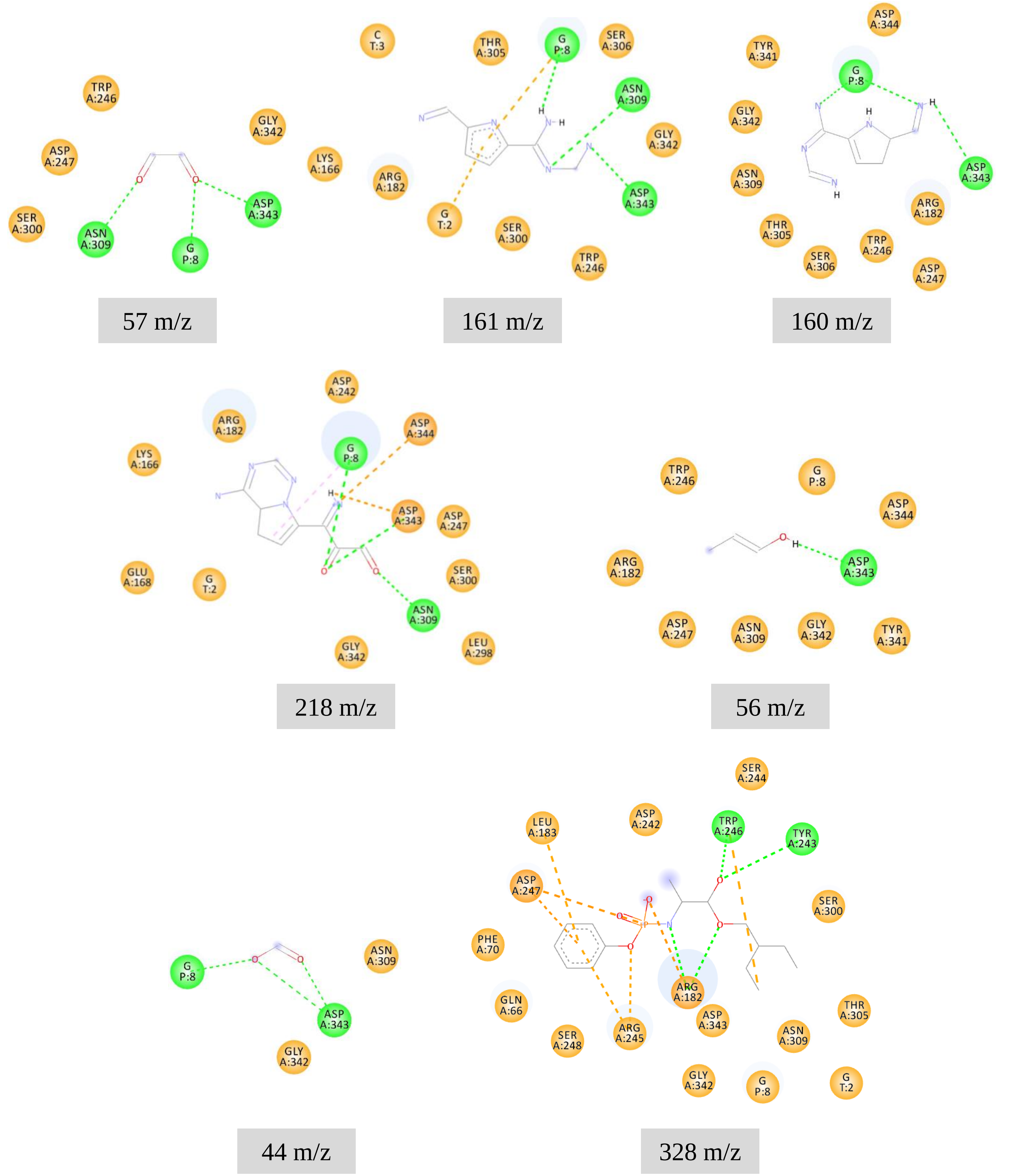

Figure S.6.7-a. Intermolecular interactions of the fragments of Remdesivir in the RdRp binding site. Green = hydrogen bond, Orange = hydrophobic interaction.

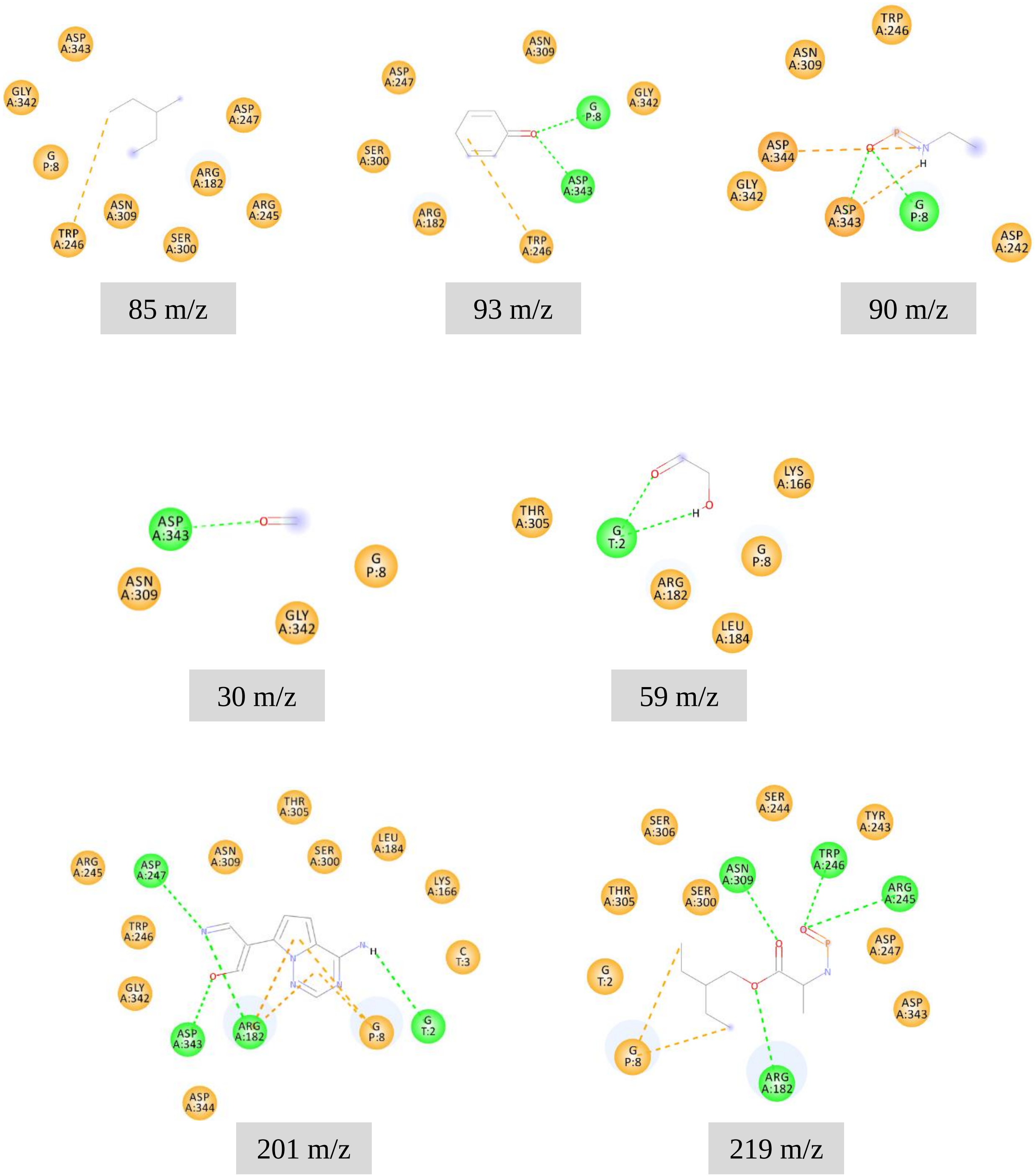

Figure S.6.7-b. Intermolecular interactions of the fragments of Remdesivir in the RdRp binding site. Green = hydrogen bond, Orange = hydrophobic interaction.

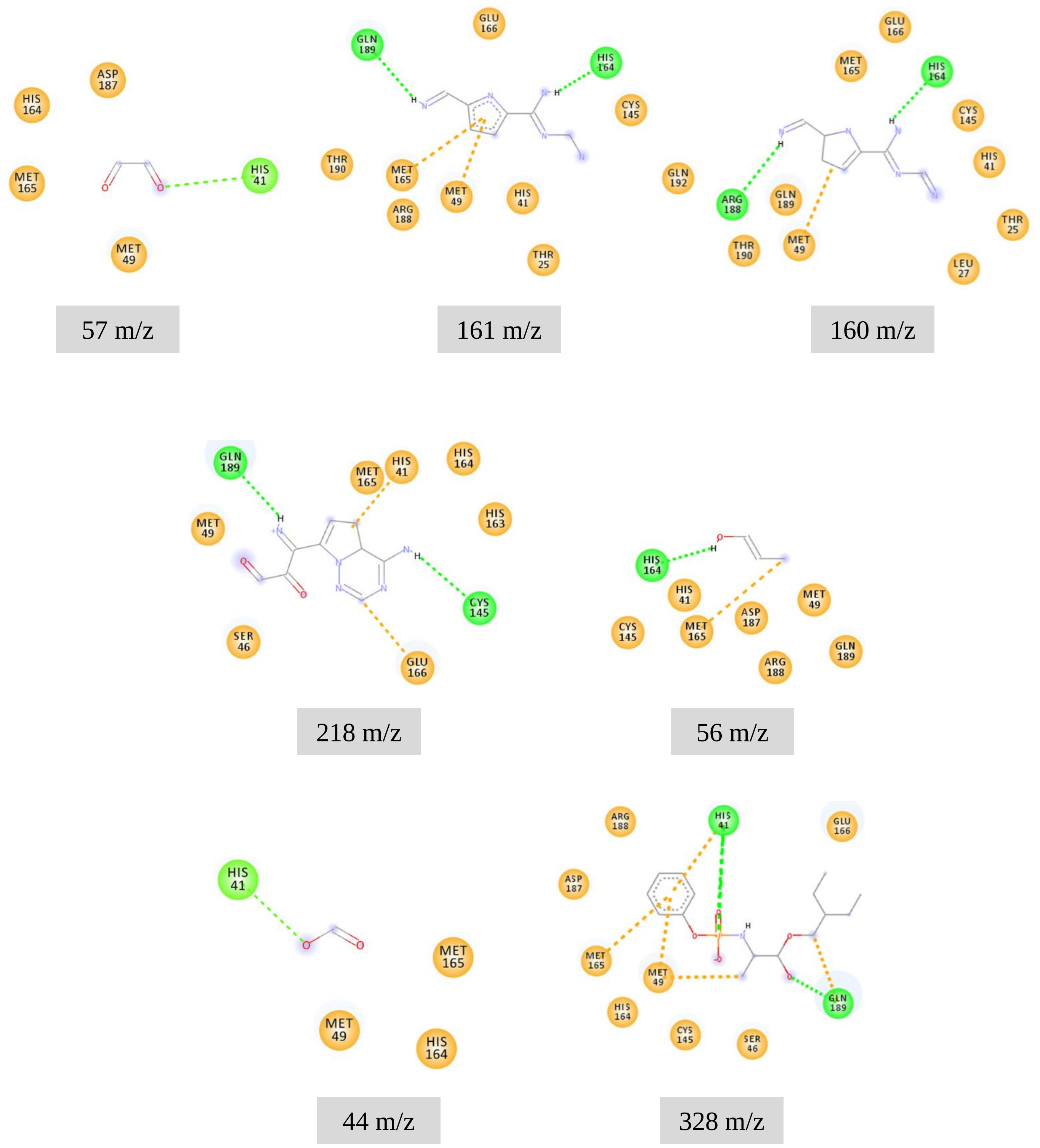

Figure S.6.8-a. Intermolecular interactions of the fragments of Remdesivir in the M^{pro} binding site. Green = hydrogen bond, Orange = hydrophobic interaction.

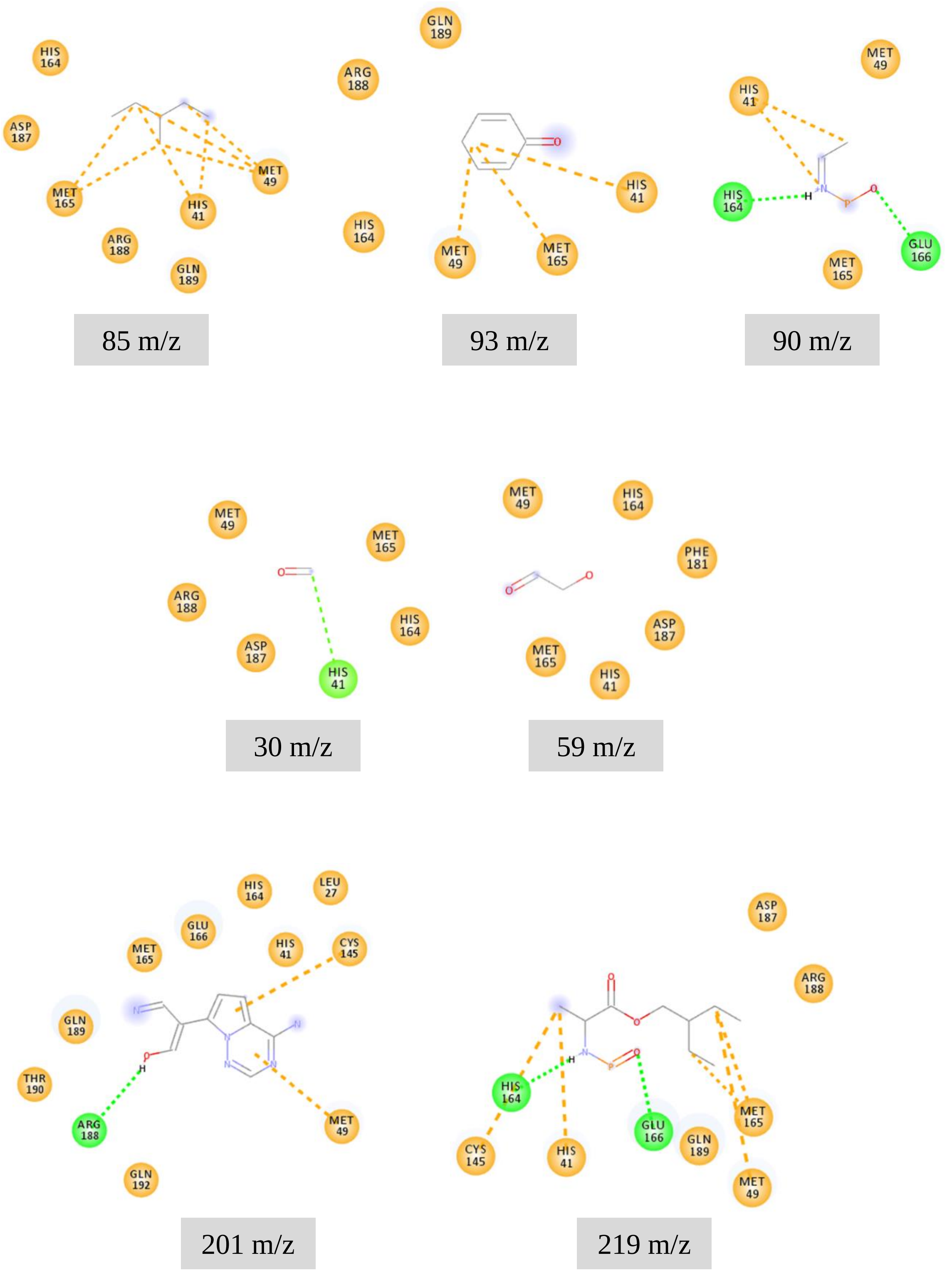

Figure S.6.8-b. Intermolecular interactions of the fragments of Remdesivir in the M^{pro} binding site. Green = hydrogen bond, Orange = hydrophobic interaction.

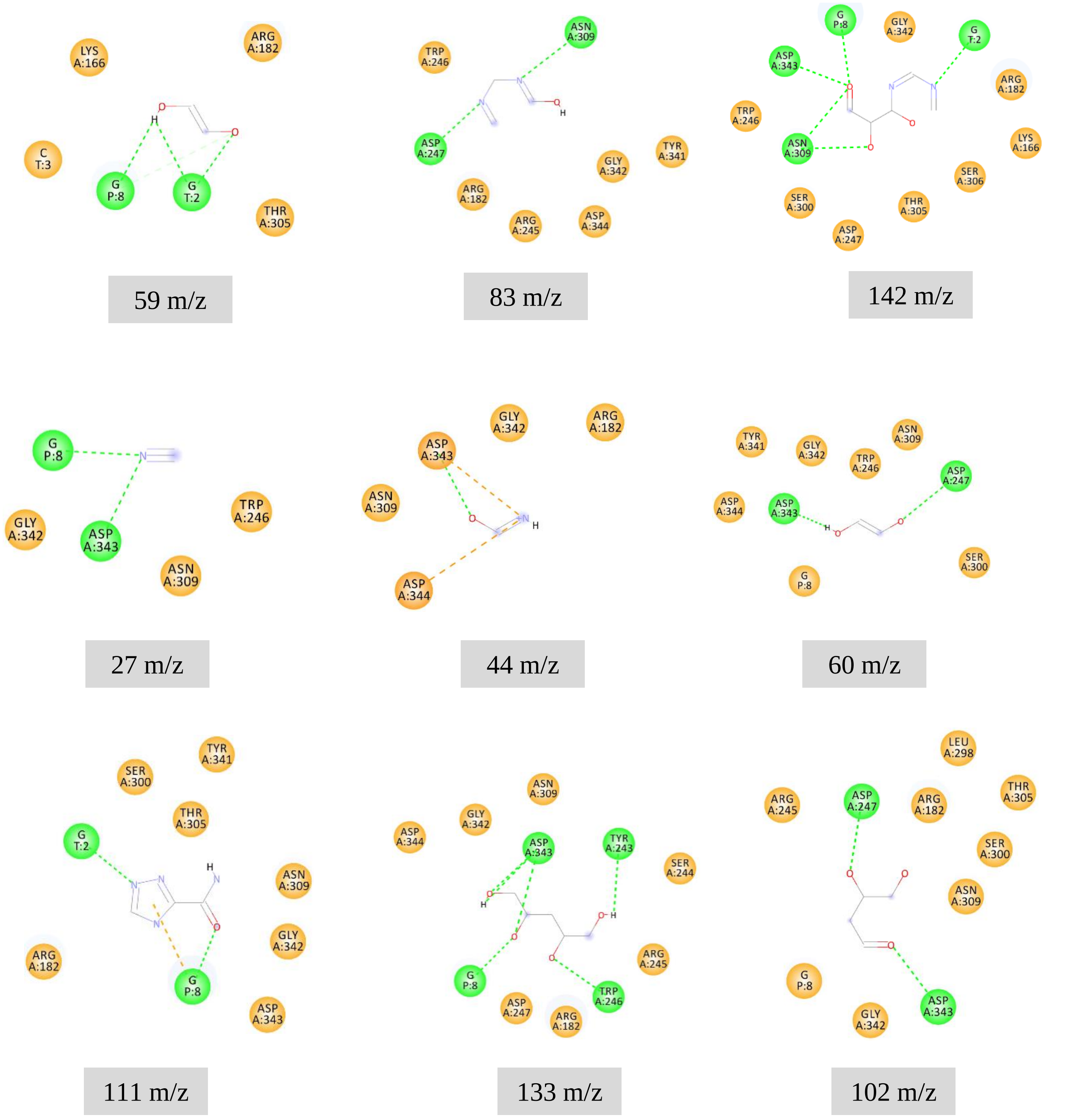

Figure S.6.9. Intermolecular interactions of the fragments of Ribavirin in the RdRp binding site. Green = hydrogen bond.

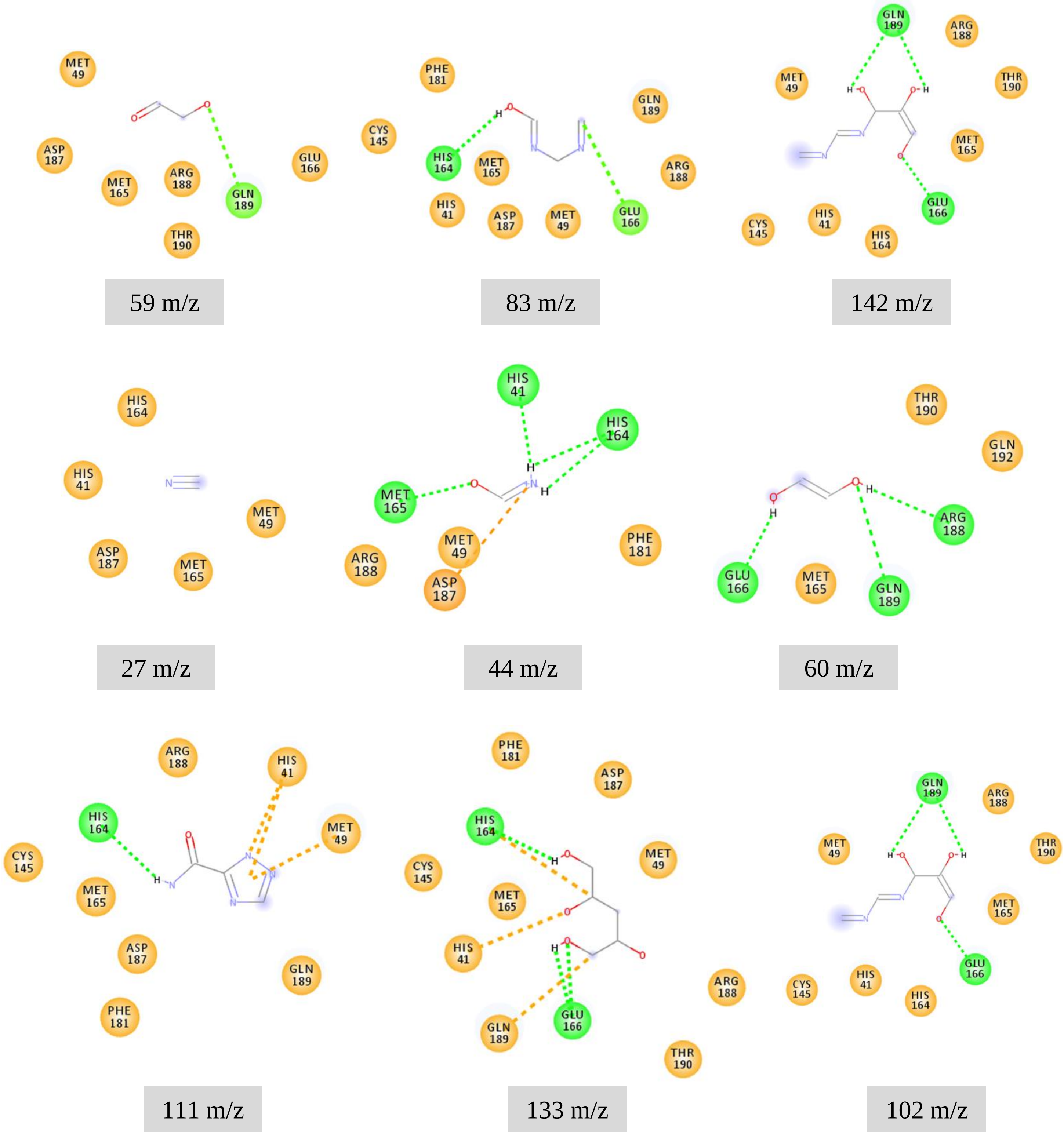

Figure S.6.10. Intermolecular interactions of the fragments of Ribavirin in the M^{pro} binding site. Green = hydrogen bond, Orange = hydrophobic interaction.

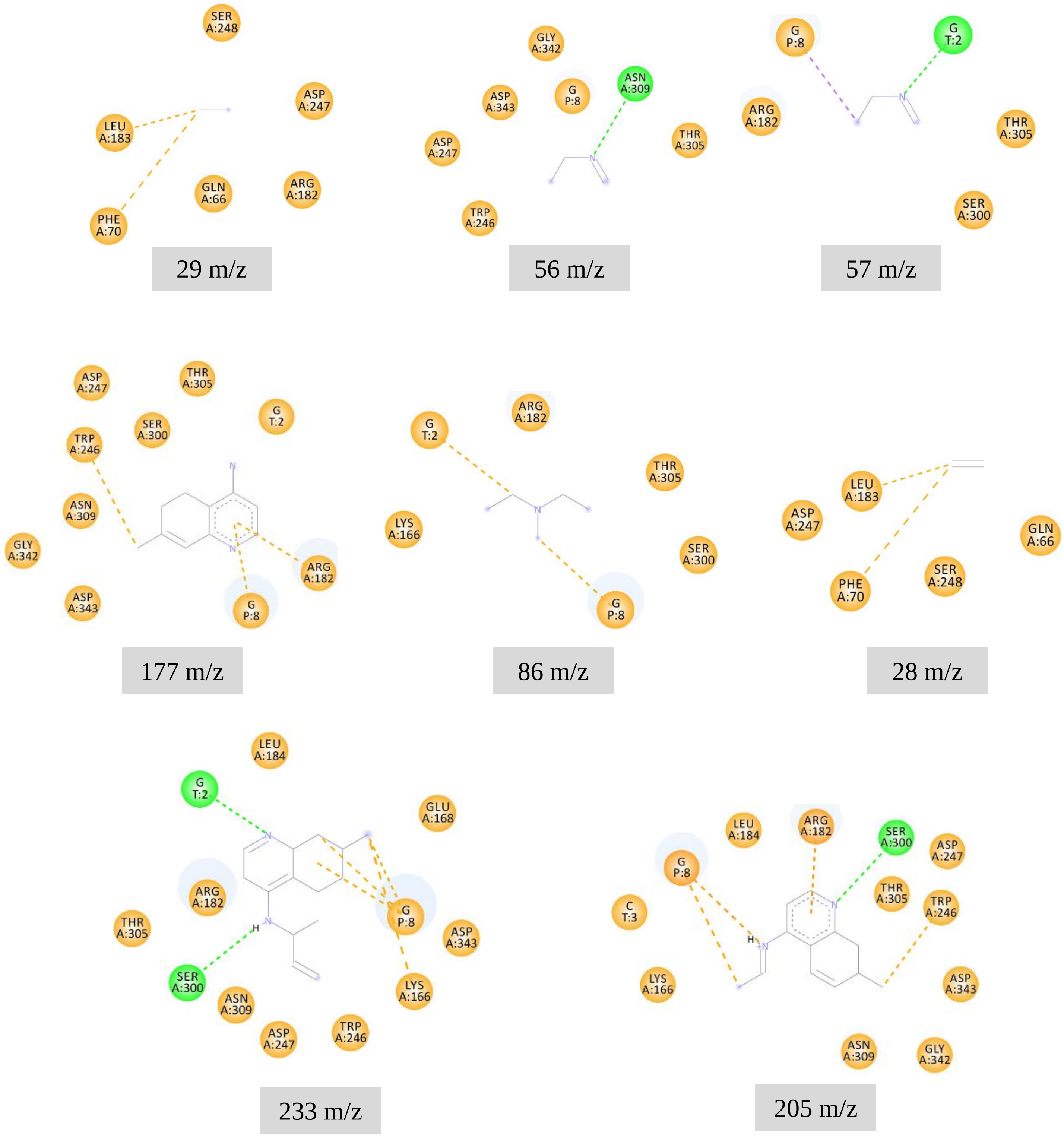

Figure S.6.11. Intermolecular interactions of the fragments of Chloroquine in the RdRp binding site. Green = hydrogen bond, Orange = hydrophobic interaction.

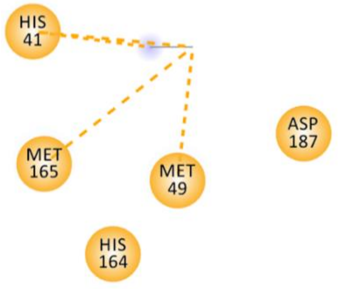

29 m/z

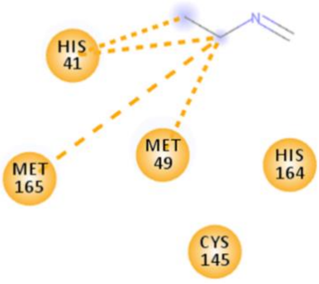

56 m/z

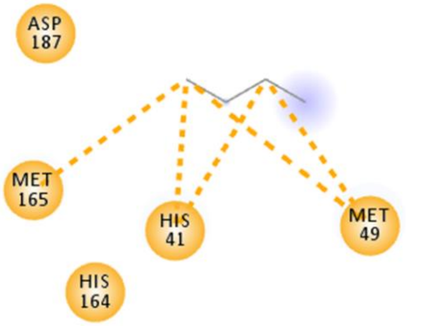

57 m/z

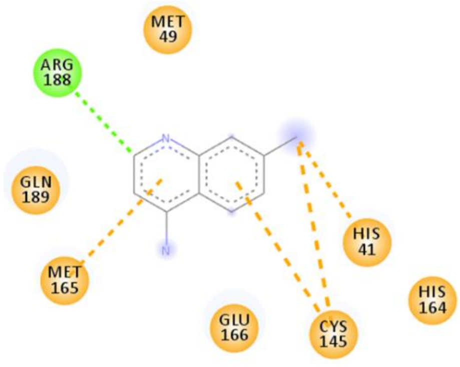

177 m/z

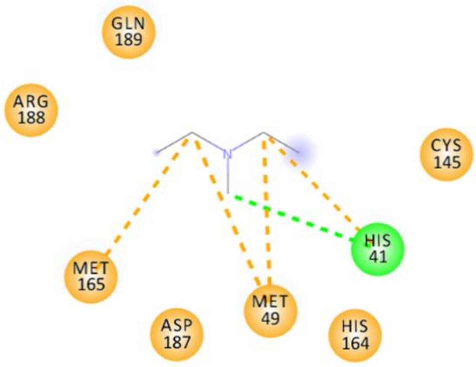

86 m/z

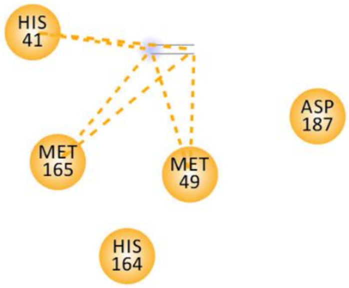

28 m/z

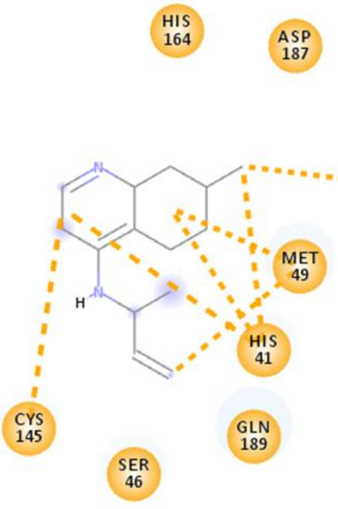

233 m/z

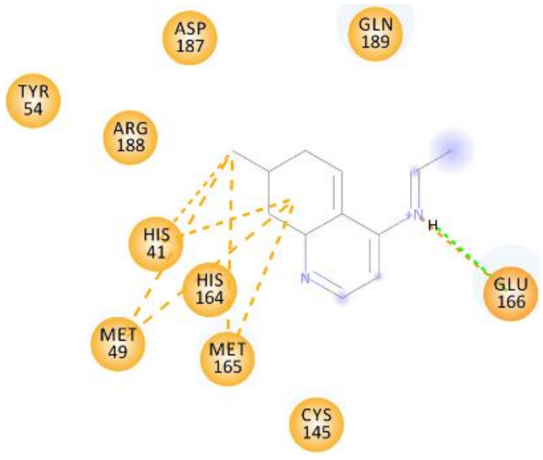

205 m/z

Figure S.6.12. Intermolecular interactions of the fragments of Chloroquine in the M^{pro} binding site. Green = hydrogen bond, Orange = hydrophobic interaction.

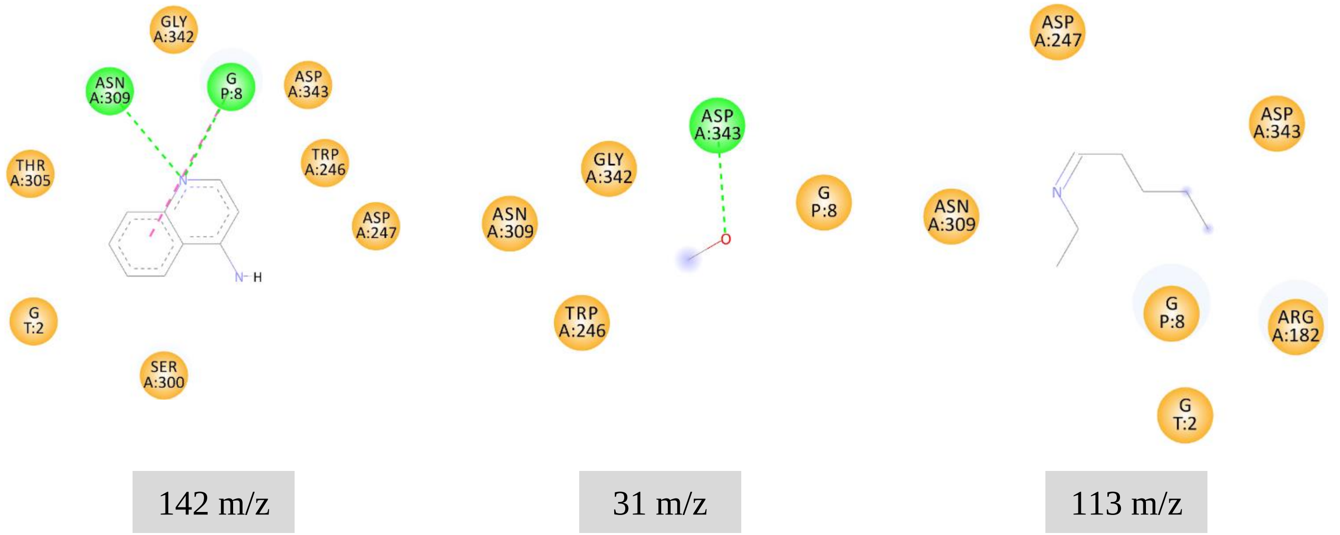

Figure S.6.13. Intermolecular interactions of the fragments of Hydroxychloroquine in the RdRp binding site. Green = hydrogen bond.

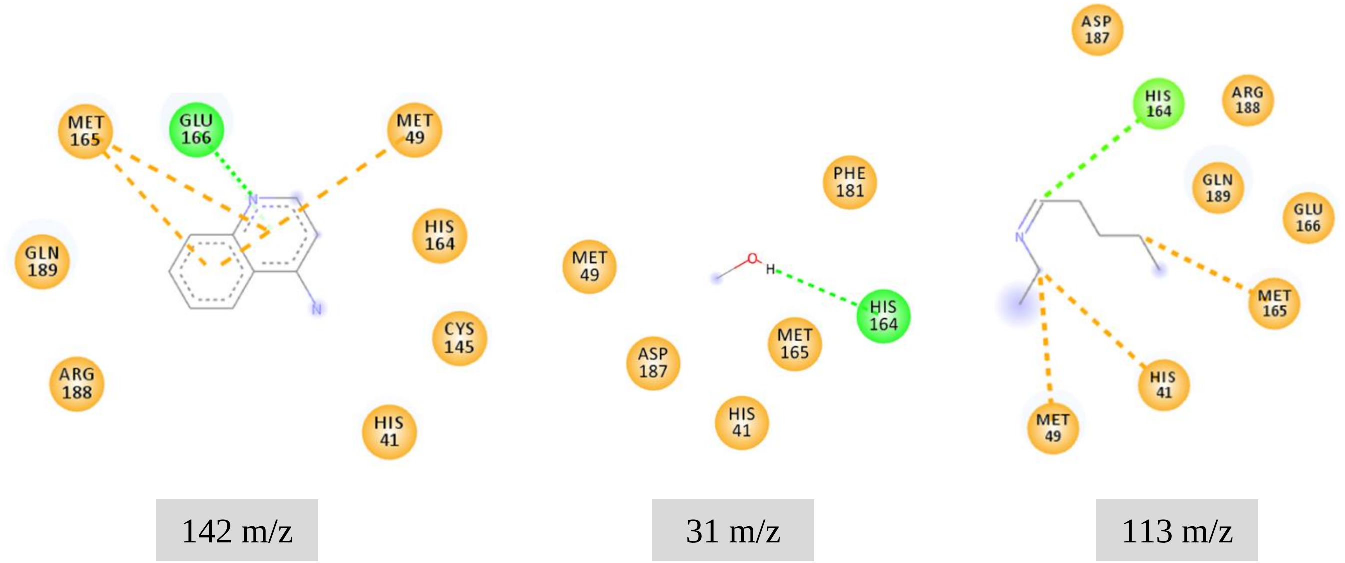

Figure S.6.14. Intermolecular interactions of the fragments of Hydroxychloroquine in the M^{pro} binding site. Green = hydrogen bond, Orange = hydrophobic interaction.

REFERENCES

1. Assis, L. C. *et al.* Nitro derivatives of quinoline and quinoline N-oxide as low-cost alternative for the treatment of SARS-CoV-2 infection. (2020) doi:10.21203/rs.3.rs-32468/v1.
2. Castro, A. de, Assis, L., Ramalho, T. & Porta, F. La. New in silico insights into the application of the (hydroxy)chloroquine with macrolide antibiotics co-crystals against the SARS-CoV-2 virus. (2020) doi:10.21203/rs.3.rs-66640/v1.
3. Ducharme, J. & Farinotti, R. Clinical Pharmacokinetics and Metabolism of Chloroquine. *Clin. Pharmacokinet.* **31**, 257–274 (1996).
4. Ette, E. I., Essien, E. E., Thomas, W. O. A. & Brown-Awala, E. A. Pharmacokinetics of Chloroquine and Some of Its Metabolites in Healthy Volunteers: A Single Dose Study. *J. Clin. Pharmacol.* **29**, 457–462 (1989).
5. McChesney, E. W., Banks, W. F. & Sullivan, D. J. Metabolism of chloroquine and hydroxychloroquine in albino and pigmented rats. *Toxicol. Appl. Pharmacol.* **7**, 627–636 (1965).
6. Reingruber, H. & Pontel, L. B. Formaldehyde metabolism and its impact on human health. *Curr. Opin. Toxicol.* **9**, 28–34 (2018).
7. Eells, J. T. Formaldehyde Poisoning. *JAMA* **246**, 1237 (1981).
